# Multi‐organ single‐cell RNA sequencing in mice reveals early hyperglycemia responses that converge on fibroblast dysregulation

**DOI:** 10.1096/fj.202302003R

**Published:** 2024-02-02

**Authors:** Adam T. Braithwaite, Naveed Akbar, Daniela Pezzolla, Daan Paget, Thomas Krausgruber, Christoph Bock, Ricardo Carnicer, Robin P. Choudhury

**Affiliations:** ^1^ Division of Cardiovascular Medicine, Radcliffe Department of Medicine University of Oxford Oxford UK; ^2^ CeMM Research Center for Molecular Medicine of the Austrian Academy of Sciences Vienna Austria; ^3^ Medical University of Vienna Institute of Artificial Intelligence, Center for Medical Data Science Vienna Austria

**Keywords:** endothelial cells, fibrosis, gene expression, hyperglycemia, mice, monocytes, myofibroblast, RRID:IMSR_JAX:000664, RRID:SCR_001618, RRID:SCR_001905, RRID:SCR_007322, RRID:SCR_019010, RRID:SCR_021946, RRID:SCR_022146, RRID:SCR_022254, single‐cell RNA‐seq, streptozotocin, type 1 diabetes

## Abstract

Diabetes causes a range of complications that can affect multiple organs. Hyperglycemia is an important driver of diabetes‐associated complications, mediated by biological processes such as dysfunction of endothelial cells, fibrosis, and alterations in leukocyte number and function. Here, we dissected the transcriptional response of key cell types to hyperglycemia across multiple tissues using single‐cell RNA sequencing (scRNA‐seq) and identified conserved, as well as organ‐specific, changes associated with diabetes complications. By studying an early time point of diabetes, we focus on biological processes involved in the initiation of the disease, before the later organ‐specific manifestations had supervened. We used a mouse model of type 1 diabetes and performed scRNA‐seq on cells isolated from the heart, kidney, liver, and spleen of streptozotocin‐treated and control male mice after 8 weeks and assessed differences in cell abundance, gene expression, pathway activation, and cell signaling across organs and within organs. In response to hyperglycemia, endothelial cells, macrophages, and monocytes displayed organ‐specific transcriptional responses, whereas fibroblasts showed similar responses across organs, exhibiting altered metabolic gene expression and increased myeloid‐like fibroblasts. Furthermore, we found evidence of endothelial dysfunction in the kidney, and of endothelial‐to‐mesenchymal transition in streptozotocin‐treated mouse organs. In summary, our study represents the first single‐cell and multi‐organ analysis of early dysfunction in type 1 diabetes‐associated hyperglycemia, and our large‐scale dataset (comprising 67 611 cells) will serve as a starting point, reference atlas, and resource for further investigating the events leading to early diabetic disease.

## Graphical abstract

The study presents a multi‐organ (heart, liver, kidney, and spleen) single‐cell transcriptomic analysis of hyperglycemia in streptozotocin‐treated and healthy mice. Patterns of gene expression were assessed in key cell types: endothelial cells, fibroblasts, monocytes, and macrophages. Distinct responses were noted in most cells between organs, while fibroblasts had many common features, relating to their potential origin and genes associated with metabolic and inflammatory activity. Created with BioRender.com.

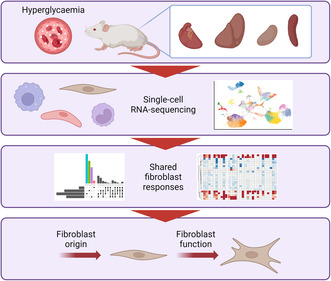

**Abbreviations**
EC: endothelial cell
ECM: extracellular matrix
EMT: epithelial‐to‐mesenchymal transition
EndoMT: endothelial‐to‐mesenchymal transition
FBS: fetal bovine serum
HTO: hashtag oligo
RAGE: receptor for advanced glycation end products
scRNA‐seq: single‐cell RNA sequencing
STZ: streptozotocin
tSNE: t‐distributed stochastic neighbor embedding
UMI: Unique Molecular Identifiers

## 1 INTRODUCTION

Hyperglycemia is central to the diagnosis, monitoring, and treatment in patients with both type 1 and type 2 diabetes. Exposure to blood glucose elevation is shared by all organs and tissues, but the loss of function leading to clinical manifestations is, naturally, organ specific. However, some underlying molecular and cellular pathologies could be shared. For instance, high glucose levels can exacerbate disease^1^ by initiating the nonenzymatic glycation of proteins and lipoproteins, leading to activation of the receptor for advanced glycation end products (RAGE),^1^, ^2^ by driving oxidative stress^2^, ^3^ by promoting inflammation.^3^ Several cell types contribute to the progression of diabetes‐associated complications and include dysfunction of endothelial cells (ECs),^4^ fibrosis/deposition of extracellular matrix (ECM) by fibroblasts,^5^ and alterations in generation of leukocytes, and their programming and recruitment.^6^, ^7^, ^8^

Alterations in cellular differentiation and functional states are observed in many different diseases and contribute to diabetes‐associated complications. For example, activated, pro‐fibrotic cells can be derived from other transitioning cell types via a biological process called epithelial‐to‐mesenchymal transition (EMT), where a polarized epithelial cell, which normally interacts with basement membrane via its basal surface, assumes a mesenchymal cell phenotype, characterized by enhanced migratory capacity, invasiveness, resistance to apoptosis, and greatly increased production of ECM components. Similarly, circulating bone marrow‐derived myeloid cells, such as monocytes or macrophages, have been shown to transition into fibrocytes or fibroblasts that contribute to the development of fibrosis‐related complications of diabetes.^9^, ^10^ Furthermore, in endothelial‐to‐mesenchymal transition (EndoMT), an EC undergoes a series of molecular events that change its phenotype toward a mesenchymal cell with myofibroblast or smooth muscle cell function. EndoMT causes loss of tight junctions in glomeruli leading to increased endothelial permeability and albuminuria.^11^, ^12^

Given the potential for shared early cellular pathologies secondary to hyperglycemia, and the importance of ECs, fibroblasts, macrophages, and monocytes across organs affected by these pathologies, we hypothesized that processes of disease initiation might be shared between organs, with subsequent divergence and clinical manifestation according to organ‐specific composition and function. Accordingly, we comprehensively characterized cell populations at an early time point prior to overt pathology, using single‐cell RNA‐sequencing (scRNA‐seq) profiles in four organs (heart, kidney, liver, and spleen) associated with diabetic complications using the streptozotocin (STZ) model of hyperglycemia in mice. scRNA‐seq is a powerful approach for determining cellular composition and transcriptional phenotypes, allowing comparisons between source conditions such as developmental stage, organ of origin, and pathological state.^13^ While several studies have utilized this technology to characterize cells from affected organs individually; particularly the kidney,^14^, ^15^ none, to our knowledge have attempted an integrated systematic analysis of hyperglycemia‐related changes across disease‐relevant organs.

Our study uncovered changes in cellular developmental and functional programming with plausible roles in the initiation of the complications of diabetes. Overall, we identified organ‐specific as well as shared pathological mechanisms, providing potential targets for preventative and therapeutic interventions for diabetes‐associated complications.

## 2 MATERIALS AND METHODS

### 2.1 Animals

All animal protocols were conducted in accordance with the UK Home Office under the Guidance of the Operation of the Animals (Scientific Procedures) Act 1986, complying with all ethical regulations and the institutional review board guidelines (project license 30/3374; January 27, 2016). Wild‐type C57BL/6J (RRID:IMSR_JAX:000664) (12‐ to 14‐week‐old) male mice were randomly assigned to control or STZ experimental groups. Diabetes was induced by intraperitoneal injection of low‐dose (42–45 mg·kg^−1^·d^−1^) STZ over 5 consecutive days. At 8 weeks after the final streptozotocin injection, nonfasted blood glucose was measured, animals were euthanized, and tissue was collected. Blood glucose levels were between 11.3 and 35.0 mmol/L (mean 22.3 mmol/L) for the STZ mice and 9.0 and 10.1 mmol/L (mean 8.4 mmol/L) for the control mice. There was no lethality within either experimental group. Tissues from *n* = 3 control mice and *n* = 4 STZ mice (with blood glucose levels >20 mmol/L) were used for scRNA‐seq.

### 2.2 Cell isolation

Heart (ventricle), liver, and kidneys were dissected and finely minced on ice using scissors. Cells were enzymatically dissociated in collagenase II (500 U/mL; Thermo Fisher 17 101 015) in 10 mL HBSS, with agitation at 37°C for 1 hour. Dissociated heart, liver, and kidney cells were passed through a 70 μm cell strainer and rinsed with 3 mL ice‐cold HBSS. Spleens were passed through a 70 μm cell strainer into 1X red cell lysis buffer (1.5 mM ammonium chloride, 100 μM sodium bicarbonate, and 11 μM EDTA disodium) and incubated for 10 minutes at room temperature. All cells were centrifuged for 5 min at 400*g* at 4°C, then resuspended in 10% v/v DMSO in fetal bovine serum (FBS), aliquoted, and frozen at −80°C before sequencing.

### 2.3 Single‐cell RNA sequencing

Cells were revived by thawing at 37°C for 1 min in a water bath and slowly transferred into 10 mL tubes filled with 9 mL pre‐warmed RPMI containing 10% FBS and centrifuged at 500*g* for 5 min. Next, cells were resuspended in 500 μL RPMI containing 10% FBS and labeled with commercially available DNA‐labeled antibodies (TotalSeq‐A, Biolegend) at 4°C for 30 min. Following incubation, cell suspensions were washed three times with PBS containing 2% BSA. After the final wash, cell suspensions were resuspended in PBS containing 2% BSA and a viability dye (Zombie Red, Biolegend) for discrimination between live and dead cells. Next, 10 000 viable cells were sort purified from the four organs of each individual mouse using an SH800 cell sorter (Sony). The four cell fractions were pooled for processing as a single sample according to the manufacturer's protocol, while the antibody‐linked barcodes enabled sample‐specific demultiplexing of the sequencing data. scRNA‐seq libraries were generated using the chromium controller and the next GEM Single‐Cell 3’ Reagent Kit (v3, 10x Genomics) according to the manufacturer's instructions. Libraries were sequenced by the Biomedical Sequencing Facility at the CeMM Research Center for Molecular Medicine of the Austrian Academy of Sciences, using the Illumina NovaSeq 6000 platform.

### 2.4 Single‐cell library analysis

Reads from Unique Molecular Identifiers (UMIs) were aligned and counted per gene with 10x Genomics Cell Ranger 5.0.1,^16^ and the Seurat 4.1.0 R‐package (http://seurat.r‐forge.r‐project.org/, RRID:SCR_007322)^17^, ^18^, ^19^ was used for hashtag oligo (HTO) demultiplexing and doublet removal. Low‐quality and potential doublet cells were excluded by presence of two or more HTO tissue assignments, high percent mitochondria (>25%), number of genes detected (<300 or >5000), and number of UMIs detected (>25 000). Normalization was performed for each sample with sctransform 0.3.3 (https://github.com/satijalab/sctransform, RRID:SCR_022146)^20^ using the gamma‐Poisson method provided by glmGamPoi 1.8.0 (https://bioconductor.org/packages/glmGamPoi/),^21^ with regression for percent mitochondria and predicted cell cycle difference (i.e., S‐score minus G2M‐score).

### 2.5 Integrated single‐cell analysis

All single‐cell transcriptome profiles were integrated with Seurat using the “SCT” method to minimize batch effects. Variation of gene expression across cells was assessed by PCA and then UMAP dimensional reduction based on the first 30 dimensions. Cell clusters were determined using Seurat with a resolution of 0.8, which was determined to be optimal for cluster resolution and stability. Next, we used the Seurat “FindConservedMarkers” function to identify marker genes of the individual clusters that were conserved across STZ and control groups. Cell types were annotated based on marker genes with a combined iterative approach including manual database searches of Tabula Muris (https://tabula‐muris.ds.czbiohub.org/)^22^ and the Mouse Cell Atlas (https://bis.zju.edu.cn/MCA/),^23^ literature searches and the automated tools scCATCH 3.1 (https://github.com/ZJUFanLab/scCATCH),^24^ and SingleR 1.10.0 (https://www.bioconductor.org/packages/release/bioc/html/SingleR.html, RRID:SCR_023120).^25^ Pseudo‐bulk RNA‐seq profiles were generated by summing reads per gene across all cells per sample followed by calculating gene expression with DESeq2 (https://bioconductor.org/packages/release/bioc/html/DESeq2.html, RRID:SCR_015687) 1.36.0^26^ and normalizing across genes with a variance stabilizing transformation.

### 2.6 Interdisease group single‐cell comparisons

Differential gene expression was assessed between STZ and control cells of each general cell type per organ using the “FindMarkers” function of Seurat with the Wilcoxon rank‐sum test, with significant adjusted p‐values determined at *p* < .05. All genes from each comparison were ranked by sign(log2FC) × −log10(*p*‐value) and evaluated using gene‐set enrichment analysis with fGSEA 1.22.0 (https://bioconductor.org/packages/fgsea/, RRID:SCR_020938)^27^ to determine enrichment of mouse‐mapped versions of the Reactome pathway annotation set (version 0.3) from MSigDB (http://software.broadinstitute.org/gsea/msigdb/index.jsp, RRID:SCR_016863),^28^ limited to pathways with <200 genes. Enriched gene sets were collapsed to remove nonindependent gene sets. Over‐representation analysis was performed using the hypergeometric test within the fGSEA package for distinct sets of up‐regulated and down‐regulated differential genes (log2FC >0.1, *p* < .05) per tissue/cell type (for those with >150 cells) and for shared genes between cells of combinations of tissues. Cell signaling within each tissue was assessed by expression of ligand/receptor pairs using CellChat 1.0.0 (https://github.com/sqjin/CellChat, RRID:SCR_021946)^29^ with adjustment for the effect of cell population size.

### 2.7 Mendelian randomization analysis

Two‐sample Mendelian randomization analyses were carried out using the TwoSampleMR R‐package provided by MR‐Base (https://github.com/MRCIEU/TwoSampleMR, RRID:SCR_019010).^30^, ^31^ Instrumental variables were eQTL loci from the GTEx database (http://www.ncbi.nlm.nih.gov/gtex/GTEX2/gtex.cgi, RRID:SCR_001618)^32^ and outcomes were T1D traits from the FinnGen GWAS study (https://finngen.gitbook.io/documentation/, RRID:SCR_022254).^33^

### 2.8 Statistical analyses

Statistical analyses and visualizations were performed with R 4.1.1 (http://www.r‐project.org/, RRID:SCR_001905). The significance threshold for all statistical testing was set at *p* < .05 and appropriate adjustment for multiple testing (Benjamini–Hochberg or Bonferroni) was utilized for all analyses.

## 3 RESULTS

### 3.1 Multi‐organ single‐cell transcriptome profiles from hyperglycemic and control mice

To study cell composition and gene regulation in diabetes, we used the STZ mouse model of hyperglycemia and specifically focused on an early timepoint, that is, after induction of hyperglycemia but before the onset of macroscopic pathology, to investigate and characterize the initiation of diabetes‐related tissue pathology. STZ administration for 5 consecutive days led to the production of nonfasted blood glucose levels (>20 mmol/L) and increased terminal glucose at the 8‐week time point (mean 26.8 ± 6.8 in STZ mice (*n* = 4) vs. 7.9 ± 0.4 mmol/L in control mice (*n* = 3), *p* = .005; Figure S1A) associated with diabetic disease. We also observed an increase in organ masses relative to body mass upon STZ treatment in the liver (mean 55.4 vs. 42.8 mg/g, *p* = .027) and kidney (mean 13.59 vs. 11.15 mg/g, *p* = .047; Figure S1C). Absolute liver mass was also significantly higher in STZ mice than control mice (mean 1649 vs. 1305 mg, *p* = .048; Figure S1D).

To unravel the gene regulatory programs across organs, we isolated cells from the heart ventricles, kidney, liver, and spleen of control and STZ‐treated mice and performed scRNA‐seq. Cell quality was assessed by taking percentage of mitochondria, number of genes detected per cell, and number of UMIs into account (Figure S2A–C). Cells were assigned to an individual tissue according to their HTO read counts, and doublets (cells with multiple HTO barcodes) were discarded during this processing step (Figure S2D–G). We integrated the data into a joint map and performed clustering according to HTO barcode levels to confirm distinct cluster formation of singlet cells, with doublet cells located between them (Figure S2H). From each mouse a total of 7902 to 11 651 singlet cells were included in the subsequent analyses, resulting in a total of 40 710 cells from STZ‐treated mice and 26 901 cells from control mice.

### 3.2 Cell type profiles are organ specific but relatively stable across conditions

**FIGURE 1:**
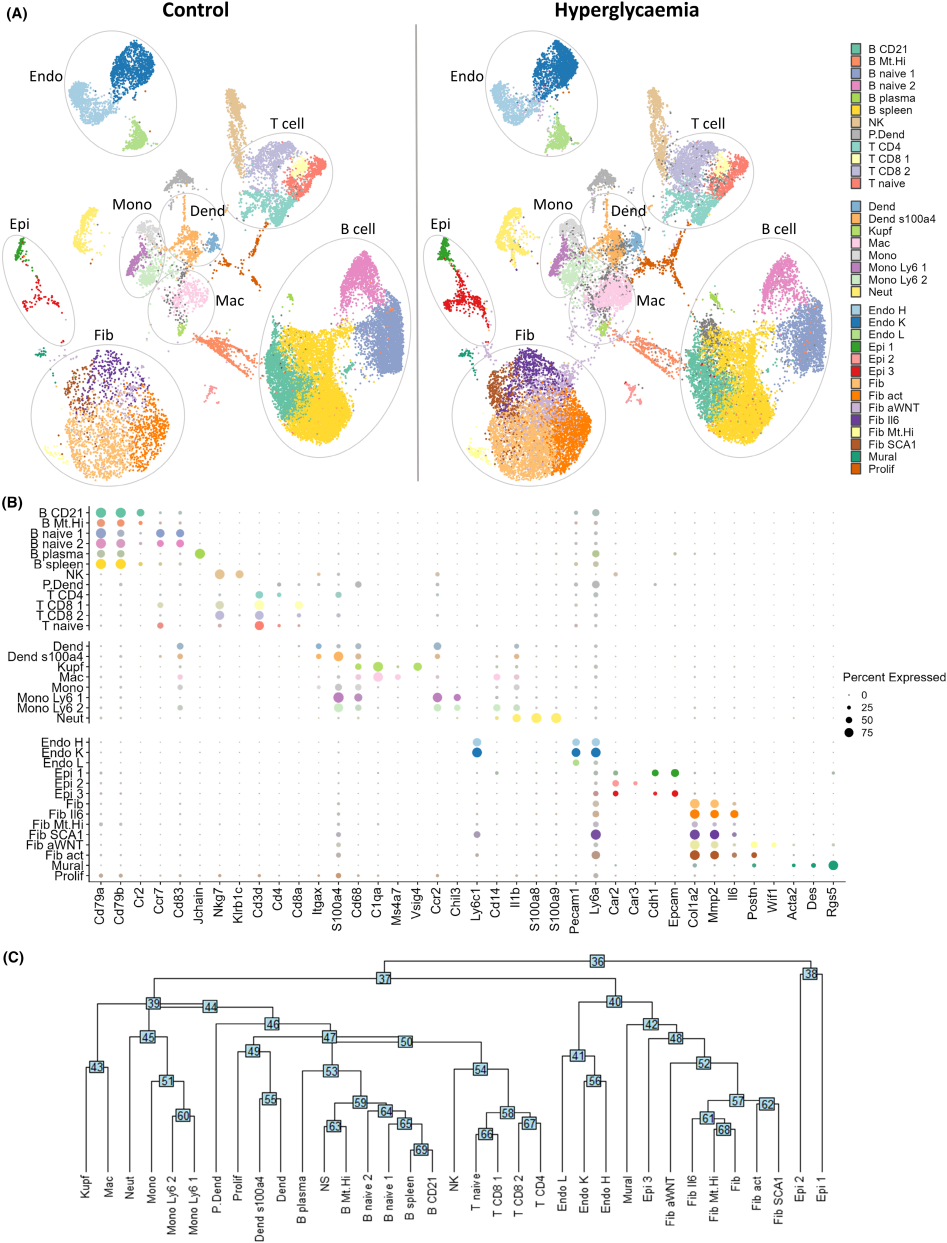
Multi‐organ single‐cell transcriptome profiles from hyperglycemic and control mice. (A) Single‐cell profiles from all organs were integrated and cells clustered. t‐distributed stochastic neighbor‐embedding (tSNE) plots show a matched number of cells from each condition. (B) Average expression of key marker genes that were used to annotate specific cell clusters and sub‐clusters. (C) The relative functional proximity of clusters was demonstrated by building a classification hierarchy with transcriptionally similar clusters adjacent to a tree. Dend, myeloid dendritic cell; Endo, endothelial cell; Epi, epithelial cell; Fib, fibroblast; Mac, macrophage; Mono, monocyte.

Single‐cell transcriptome profiles from all organs of the two experimental groups were integrated, and cells were clustered by gene expression (Figure 1A). Cell clusters were annotated based on organ specificity and differential expression of key marker genes (vs. other clusters) that were conserved across STZ and control groups (Figure 1B). The marker genes reflected well‐known cell surface proteins in addition to mRNA‐/single‐cell‐specific markers extracted from published annotations (summarized in Table 1; further details available in Supplementary Data S1). The identified cell types and cell subsets were present in the STZ and control group (Figure 1A), indicating that the treatment had no overt cell‐type‐specific effect. As expected, the cells captured primarily represented interstitial cell types due to the 10X scRNA‐seq protocol excluding larger cells such as hepatocytes or cardiomyocytes. We explored the relationships between clusters by building a classification hierarchy in which transcriptionally similar clusters assumed adjacencies on a dendrogram (Figure 1C). In accordance with the annotations, this analysis showed distinct branches for myeloid, lymphoid, and mesenchymal cell types.

**TABLE 1:** Annotation of cell types and sub‐clusters with specific marker genes.

Cell type	Organ specificity	Cell type markers	Sub‐cluster markers	Short name
B		Cd79a^+^ Cd79b^+^	Cr2^hi^ Ccr7^lo^ Cd83^lo^ Jchain^lo^ Mt^lo^	B CD21
B		Cd79a^+^ Cd79b^+^	Cr2^lo^ Ccr7^lo^ Cd83^lo^ Jchain^lo^ Mt^hi^	B Mt.Hi
B		Cd79a^+^ Cd79b^+^	Cr2^lo^ Ccr7^hi^ Cd83^hi^ Jchain^lo^ Mt^lo^	B naive 1
B		Cd79a^+^ Cd79b^+^	Cr2^lo^ Ccr7^hi^ Cd83^hi^ Jchain^lo^ Mt^lo^	B naive 2
B		Cd79a^+^ Cd79b^+^	Cr2^lo^ Ccr7^lo^ Cd83^lo^ Jchain^hi^ Mt^lo^	B plasma
B	Spleen	Cd79a^+^ Cd79b^+^	Cr2^lo^ Ccr7^lo^ Cd83^lo^ Jchain^lo^ Mt^lo^	B spleen
Dendritic		Itgax^+^	S100a4^lo^	Dend
Dendritic		Itgax^+^	S100a4^hi^	Dend s100a4
Endothelial	Heart	Pecam1^+^	Ly6a^hi^	Endo H
Endothelial	Kidney	Pecam1^+^	Ly6a^hi^	Endo K
Endothelial	Liver	Pecam1^+^	Ly6a^lo^	Endo L
Epithelial		Car2^+^	Car3^lo^ Cdh1^hi^ Epcam^hi^	Epi 1
Epithelial		Car2^+^	Car3^hi^ Epcam^lo^	Epi 2
Epithelial		Car2^+^	Car3^lo^ Cdh1^hi^ Epcam^hi^	Epi 3
Fibroblast		Col1a2^+^ Mmp2^+^	Il6^lo^ Ly6a^lo^ Postn^lo^ Wif1^lo^ Mt^lo^	Fib
Fibroblast		Col1a2^+^ Mmp2^+^	Il6^lo^ Ly6a^lo^ Postn^hi^ Wif1^lo^ Mt^lo^	Fib act
Fibroblast		Col1a2^+^ Mmp2^+^	Il6^lo^ Ly6a^lo^ Postn^hi^ Wif1^hi^ Mt^lo^	Fib aWNT
Fibroblast		Col1a2^+^ Mmp2^+^	Il6^hi^ Ly6a^lo^ Postn^lo^ Wif1^lo^ Mt^lo^	Fib Il6
Fibroblast		Col1a2^+^ Mmp2^+^	Il6^lo^ Ly6a^lo^ Postn^lo^ Wif1^lo^ Mt^hi^	Fib Mt.Hi
Fibroblast		Col1a2^+^ Mmp2^+^	Il6^lo^ Ly6a^hi^ Postn^lo^ Wif1^lo^ Mt^lo^	Fib SCA1
Macrophage		Cd68^+^ C1qa^+^	Ms4a7^hi^ Vsig4^−^	Mac
Kupffer	Liver	Cd68^+^ C1qa^+^	Ms4a7^lo^ Vsig4^+^	Kupf
Monocyte		Cd68^+^ S100a4^+^	Ccr2^lo^ Chil3^lo^ Ly6c^lo^	Mono
Monocyte		Cd68^+^ S100a4^+^	Cd14^hi^ Ccr2^hi^ Chil3^hi^ Il1b^hi^ Ly6c^hi^	Mono Ly6c 1
Monocyte		Cd68^+^ S100a4^+^	Cd14^lo^ Ccr2^hi^ Chil3^hi^ Il1b^lo^ Ly6c^hi^	Mono Ly6c 2
Mural		Acta2^+^ Des^+^ Rgs5^+^		Mural
Neutrophil		S100a8^+^ S100a9^+^		Neut
Natural killer		Nkg7^+^ Klrb1c^+^		NK
Plasmacytoid dendritic		Ccr9^+^		P. Dend
T		Cd3d^+^	Ccr7^lo^ Cd4^hi^ Cd8a^lo^ Nkg7^lo^	T CD4
T		Cd3d^+^	Ccr7^mid^ Cd4^lo^ Cd8a^hi^ Nkg7^hi^	T CD8 1
T		Cd3d^+^	Ccr7^lo^ Cd4^lo^ Cd8a^mid^ Nkg7^hi^	T CD8 2
T		Cd3d^+^	Ccr7^hi^ Cd4^hi^ Cd8a^lo^ Nkg7^lo^	T naive

Abbreviations: Hi, high; lo, low; mid, middle; Mt, (high or low) proportion of mitochondrial UMIs.

**FIGURE 2:**
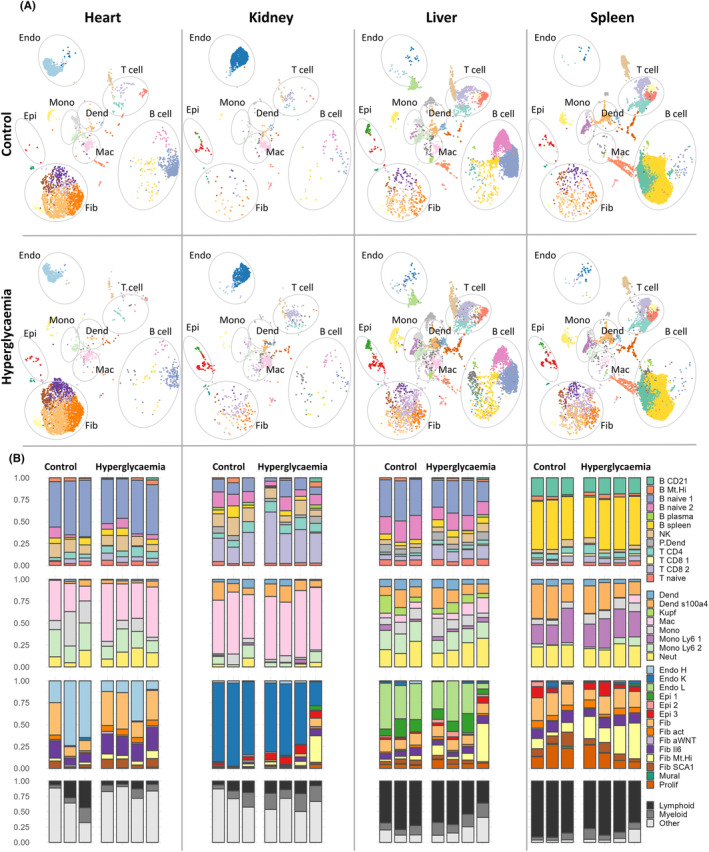
Cell‐type profiles are organ specific but relatively stable across conditions. (A) Annotated cells split by tissue of origin and group. t‐distributed stochastic neighbor‐embedding (tSNE) plots show a matched number of cells from each condition per tissue. (B) Cell sub‐clusters summarized by general type (lymphoid, myeloid, or other cells) and relative cell proportions are indicated in one column per mouse. Dend, myeloid dendritic cell; Endo, endothelial cell; Epi, epithelial cell; Fib, fibroblast; Mac, macrophage; Mono, monocyte.

Following integration and annotation, we investigated associations of scRNA‐seq profiles with organ of origin and/or hyperglycemia. Cells from all clusters and sub‐clusters were represented in the STZ and control conditions for each organ (Figure 2A). Cell clusters were summarized by general categories (lymphoid, myeloid, or other cells) to compare their relative distributions among individual mice, organs, and disease groups (Figure 2B).

**FIGURE 3:**
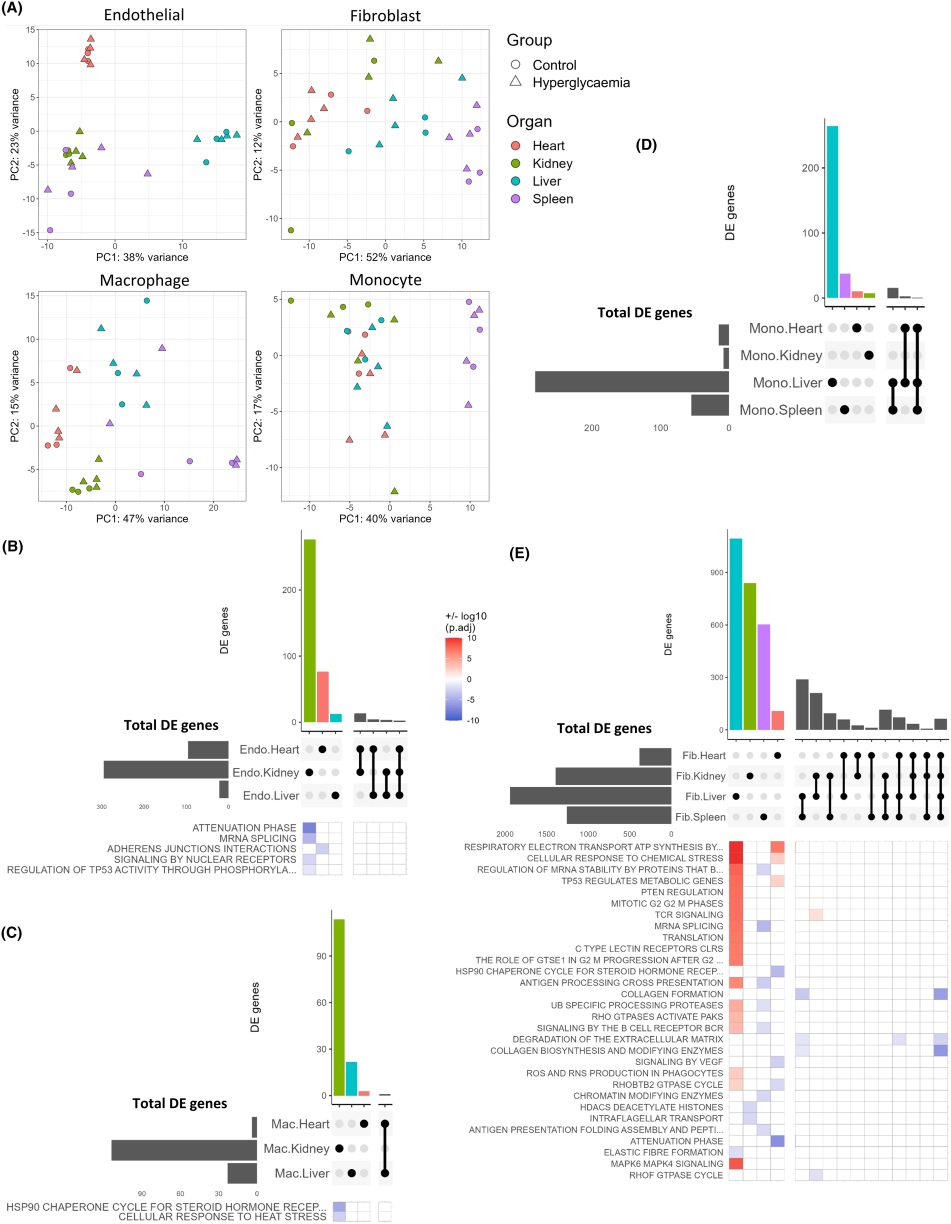
Transcriptional responses to hyperglycemia converge on fibroblasts. (A) Pseudo‐bulk RNA‐sequencing profiles were generated for each mouse/tissue/cell type by summing reads per gene per sample. The variation of gene expression for each pseudo‐bulk profile was calculated using the top 150 most variable genes and visualized for the first two principal components. Each point represents summed cells from one mouse. (B–E) For key cell types, common significant differentially expressed genes (STZ vs. control; adjusted *p* < .05 and log2FC >0.1) were assessed across organs. Upset plots show distinct and shared differential genes from (B) endothelial cells, (C) macrophages, (D), monocytes, and (E) fibroblasts, per organ. Heatmaps show +/− log10 (adjusted *p*‐value) of significantly enriched pathways from over‐representation analysis of Reactome pathways using each distinct and shared differential set of up‐ and downregulated genes (log2FC >0.1, *p* < .05) per organ (with no enriched pathways found in monocytes). Endo, endothelial cell; Fib, fibroblast; Mac, macrophage; Mono, monocyte.

To study the effect of glucose on cell‐type‐specific and organ‐specific gene regulation, we composed pseudo‐bulk RNA‐seq profiles by summing reads per gene per mouse for key cell types in each organ. Variation in gene expression for each pseudo‐bulk profile was summarized and visualized for the first two principal components showing an organ‐specific, rather than treatment‐specific, clustering for ECs, fibroblasts, macrophages, and monocytes (Figure 3A). Interestingly, organ‐specific clusters within fibroblasts were less distinct, indicating greater phenotypic similarity between organs than in other cell types.

### 3.3 Transcriptional responses to hyperglycemia converge on fibroblasts

To determine functional changes due to hyperglycemia, we performed differential expression analysis (with *p* < .05 and FC log2 ± 0.1) of cells from STZ versus control mice (Figures S3–S6). We then summarized the number of differentially expressed genes for ECs, macrophages, monocytes, and fibroblasts across organs (Figure 3B–E). In ECs, macrophages and monocytes only a minority of hyperglycemia‐responsive genes were shared across organs, indicating relative organ specificity of cellular response to hyperglycemia. However, in fibroblasts, we detected a greater proportion of differentially expressed genes shared across organs and their transcriptome profiles changed strongly in response to hyperglycemia. Shared patterns of differential gene expression from STZ‐treated mice were highest in liver and spleen fibroblasts (569 genes), followed by kidney and liver fibroblasts (455 genes).

To elucidate the biological function of the transcriptional response to hyperglycemia, we used an over‐representation analysis to annotate the Reactome pathways associated with groups of differentially expressed genes that were organ specific or shared across organs (Figure 3B–E). Several enriched pathways in STZ‐treated fibroblasts were more organ specific, including metabolism‐related terms “TP53 regulates metabolic genes” and “Respiratory electron transport, ATP synthesis by chemiosmotic coupling, and heat production by uncoupling proteins” in STZ mouse liver and heart, but not in kidney or spleen. Related to this enrichment of metabolic pathways, we also noted an increased proportion of high‐mitochondria (>10% of UMIs) fibroblasts in STZ mouse heart (Figure S7). Conversely to organ‐specific responses, pathways relating to collagen/ECM (e.g., “Collagen formation,” “Degradation of the extracellular matrix”) were associated with genes downregulated in fibroblasts from all four organs. Similar pathways were also found to be downregulated in fibroblasts using a more sensitive ranked gene set enrichment analysis (Figures S3–S6), confirming the robustness of the results. The observed pathway dysregulation was driven by lower expression of transcripts coding for collagen genes (including *Col1a1, Col4a4*, and *Col5a1*; Figures S3–S6), however, collagen genes remained among the most highly expressed transcripts within fibroblasts of both STZ and control mice. Expression of ECM‐degrading matrix metalloprotease genes (particularly *Mmp14* and *Mmp23*) was also reduced in kidney, liver, and spleen fibroblasts, but not in heart fibroblasts. Notably, the contractile marker beta‐actin (*Actb*) was upregulated in heart, kidney, liver, and spleen fibroblasts of STZ‐treated mice (*p* < .0001 in fibroblasts from each organ). In contrast, the expression of classical fibroblast markers including *Pdgfra* in liver and spleen as well as *Pdgfrb* in heart, kidney, liver, and spleen was decreased in the STZ treatment group. Overall, the transcriptional and mitochondrial differences of fibroblasts in hyperglycemia shared some features of increased metabolic activity and activation.

### 3.4 Hyperglycemia induces myeloid‐like fibroblasts

**FIGURE 4:**
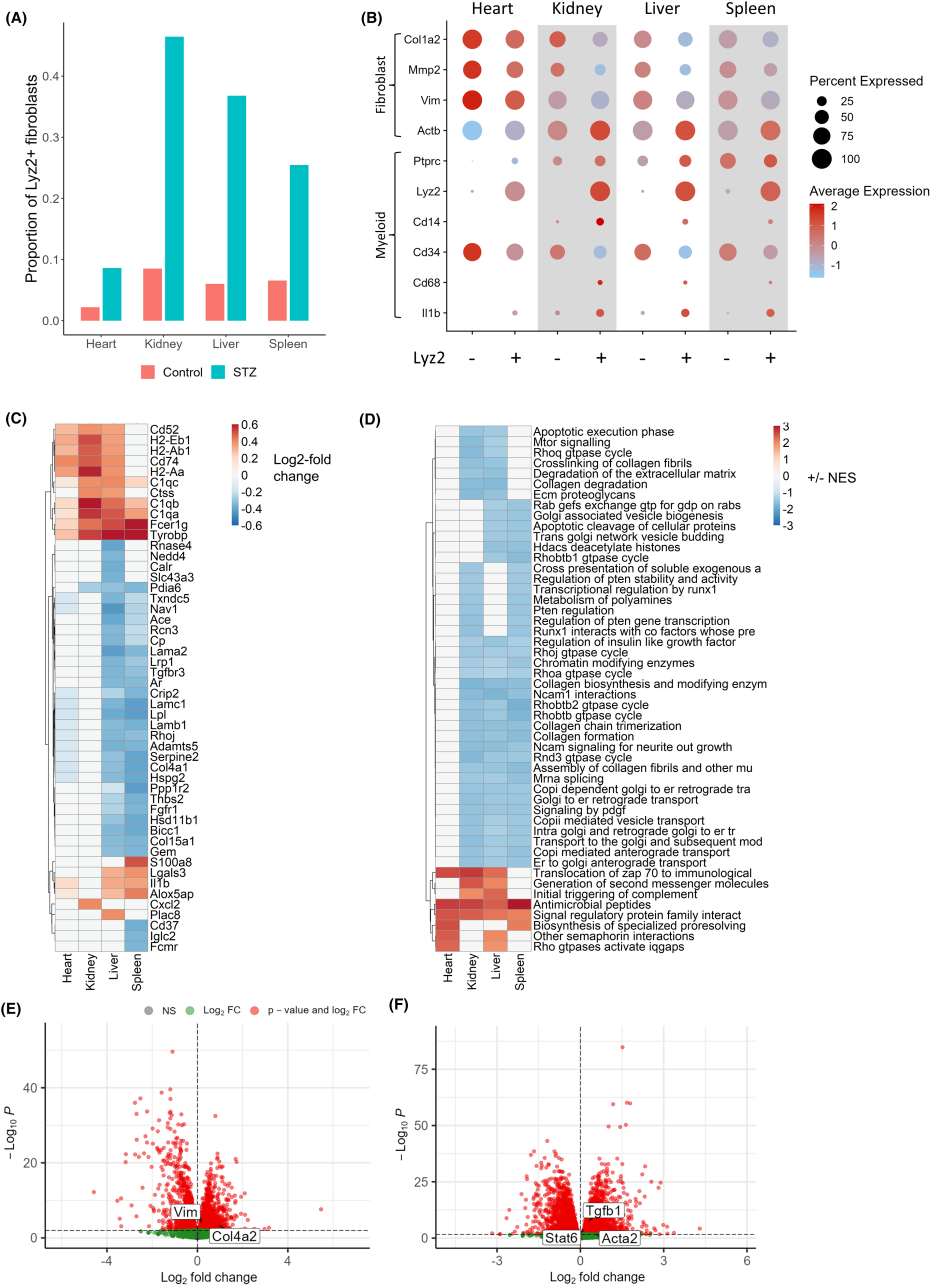
Hyperglycemia induces myeloid‐like fibroblasts. (A) Overall proportion of Lyz2^+^ fibroblasts when combining all cells from each organ/group. (B) Dot plots representing expression of key marker genes within Lyz2^+^ and Lyz2^−^ mice fibroblasts by organ. Circle color represents average expression across cells, and circle size represents the percentage of cells positive for gene expression. (C) Top 50 differentially expressed genes (when ranked by maximum log2FC) comparing Lyz2^+^ and Lyz2^−^ fibroblasts from each organ. Significantly differentially expressed genes (adjusted *p* < .05) are colored, with color representing log2FC (trimmed to ±0.6 for visualization). (D) Top 50 enriched Reactome pathways (when ranked by mean normalized enrichment score; NES) from gene set enrichment analysis of differentially expressed genes in Lyz2^+^ versus Lyz2^−^ fibroblasts. Significantly enriched pathways (adjusted *p* < .05) are colored, with color representing NES (trimmed to ±3 for visualization). Volcano plots show differentially expressed genes from bulk RNA sequencing of STZ versus control mice bone‐marrow‐derived macrophages, either (E) unstimulated or (F) stimulated with interferon‐γ and lipopolysaccharide. Key fibroblast genes are highlighted.

In addition to shared hyperglycemia‐responsive genes, we also detected higher levels of lysozyme 2 (*Lyz2*), a marker of fibroblasts/fibrocytes of circulating myeloid cell origin,^34^ in fibroblasts from the STZ treatment group. This was particularly apparent in kidney and liver, where *Lyz2* was the most highly upregulated gene (kidney, log2‐FC 2.99, *p* < .01; liver, log2‐FC 2.13, *p* < .0001). Overall, the proportion of fibroblast‐expressing *Lyz2* was increased in all organs upon STZ treatment compared to control mice (Figure 4A), with the largest disparity in kidney (46.4% vs. 8.5% *Lyz2* ^*+*^). To further characterize the *Lyz2* ^*+*^ fibroblasts, we classified all fibroblasts based on *Lyz2* expression and examined their positivity for, and expression of, key fibroblast marker genes (Figure 4B). *Lyz2* ^*+*^ fibroblasts (particularly of the kidney, liver, and spleen) had reduced positivity for *Col1a2* (e.g., 76.3% vs. 59% in liver) and *Mmp2*, but an increase in *Actb* expression (*p* < .05 in heart, *p* < .0001 in kidney and liver, and *p* < .001 in spleen), when compared to *Lyz2* ^*−*^ fibroblasts (Figure 4B). Together, these data indicate that the increased proportion of *Lyz2* ^*+*^ fibroblasts in STZ mice contributed to the apparent reduction in collagen and matrix metalloprotease gene expression (when averaged across all fibroblast populations) and corresponding increase in expression of the contractile marker beta‐actin. Notably, a greater proportion of *Lyz2* ^*+*^ fibroblasts than *Lyz2* ^*−*^ fibroblasts in kidney, liver, and spleen were positive for expression of the monocyte/macrophage marker *Cd14* (e.g., in liver, 10.6% vs. 4.4%) and macrophage marker *Cd68* (e.g., 7.1% vs. 2.8% in liver), while the proportion of *Lyz2* ^*+*^ fibroblasts (from all organs) expressing the hematopoietic stem cell marker *Cd34* was decreased compared with *Lyz2* ^*−*^ fibroblasts. A full transcriptomic differential expression analysis of *Lyz2* ^*+*^ versus *Lyz2* ^*−*^ fibroblasts revealed upregulation of complement genes (*C1qa*, *C1qb*, and *C1qc*) in fibroblasts of all four organs; MHC class II genes (*H2‐Aa*, *H2‐Ab1*, and *H2‐Eb1*) in heart, kidney, and liver fibroblasts; and the inflammatory cytokine *Il1b* in heart (*p* < .001), and liver (*p* < .0001), fibroblasts (Figure 4C). These gene expression differences were associated with differentially enriched functional pathways (Figure 4D).

To investigate whether bone marrow cells from STZ‐treated mice also display fibroblast‐like features, or a greater propensity to produce myeloid‐like fibroblasts, we examined previously generated bulk RNA‐seq data from bone‐marrow‐derived macrophages of STZ mice versus control mice.^8^ We found that unstimulated bone‐marrow‐derived macrophages from STZ mice showed an upregulation of the fibroblast markers *Vim* (*p* < .001) and *Col4a2* (*p* < .01) compared to bone‐marrow‐derived macrophages generated from control mice (Figure 4E). Furthermore, stimulation of these cells with interferon‐γ and lipopolysaccharide resulted in increased expression of genes associated with myeloid cell‐to‐fibroblast transition^9^: *Acta2* (*p* < .05), *Tgfb1* (*p* < .0001), and *Stat6* (*p* < .001), specifically in bone‐marrow‐derived macrophages from STZ‐treated mice (Figure 4F).

### 3.5 Decorin is dysregulated across cells and is linked with human type 1 diabetes

Our analysis indicated the organ of origin, rather than disease group, as the primary driver of cell‐type‐specific gene expression profiles. To investigate functional features that are shared across cell types and organs, we used gene‐set enrichment analysis and identified commonly dysregulated pathways, including ECM‐related processes (e.g., “ECM proteoglycans,” “Collagen formation,” “Extracellular matrix organization”), mRNA splicing/processing (e.g., “mRNA splicing” and “metabolism of RNA”), and heat shock function (e.g., “Cellular response to heat stress”) (Figure S8).

**FIGURE 5:**
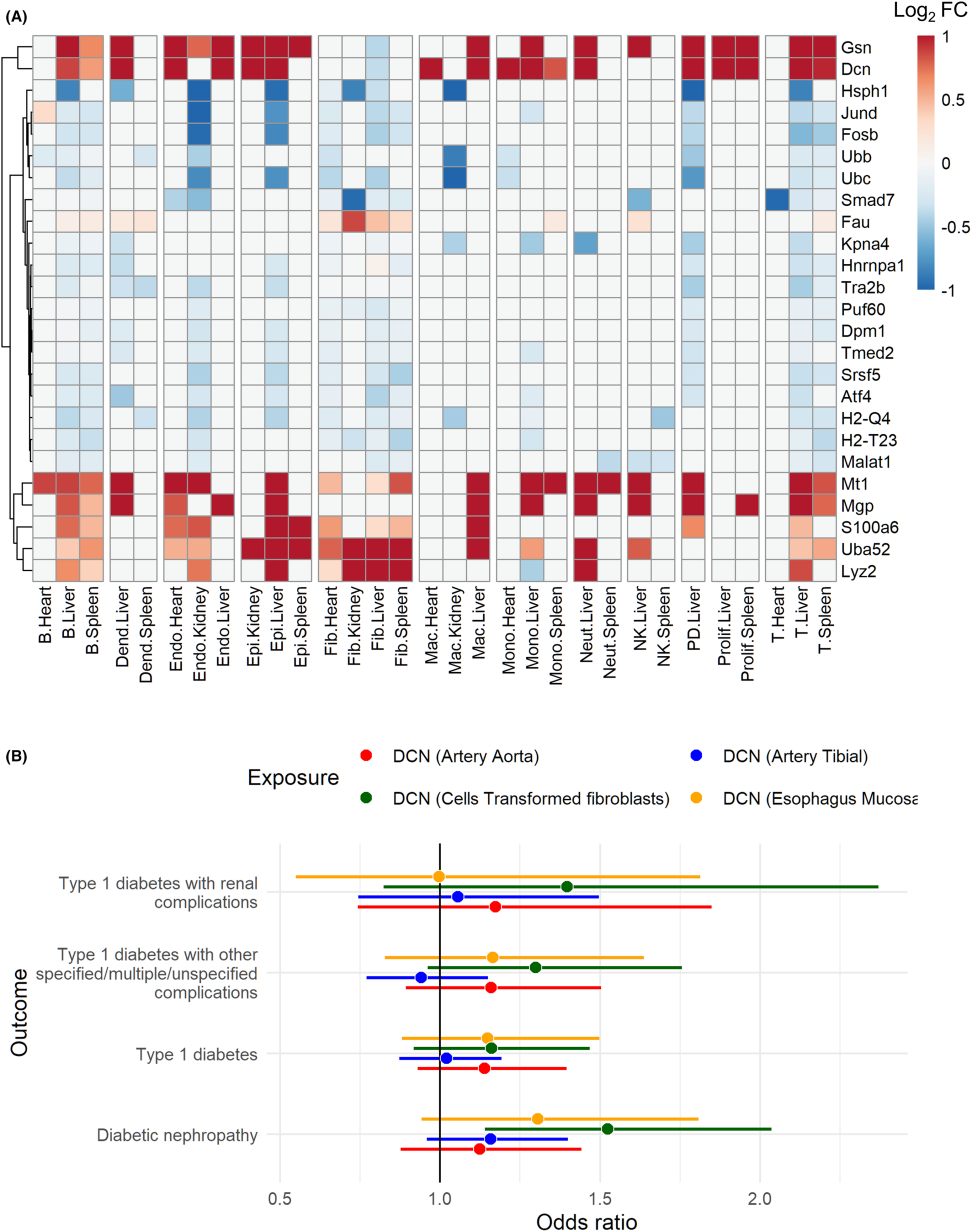
Decorin is dysregulated across cells and is linked with human type 1 diabetes. (A) The top 25 genes as ranked based on the frequency of significant differential expression (STZ vs. control) across all organ/cell types (for those with >150 total cells). Significantly differentially expressed genes (adjusted *p* < .05) are colored, with color representing log2FC (trimmed to ±1 for visualization). (B) Association of eQTLs for decorin with human type 1 diabetes traits was assessed by two‐sample Mendelian randomization. Odds ratio is plotted with bars representing lower and upper confidence intervals. DCN, decorin; Dend, myeloid dendritic cell; Endo, endothelial cell; Epi, epithelial cell; Fib, fibroblast; Mac, macrophage; Mono, monocyte; Neut, neutrophil.

We also noted that a small number of genes were commonly, and highly differentially, regulated on organ‐level analysis in STZ‐treated mice. To identify these genes, we ranked all genes based on the frequency of significant differential expression across organs and cell types of STZ‐treated mice (Figure 5A). We hypothesized that these genes might be relevant to the systemic effects of hyperglycemia, and in particular, four genes were upregulated across many cell types and organs in STZ mice: the proteoglycan decorin (*Dcn)*, gelsolin (*Gsn*), matrix Gla protein (*Mgp*), and metallothionein 1 (*Mt1*). Gelsolin is a cytoskeletal regulator of actin filament assembly/disassembly, while decorin and matrix Gla protein are both components of the extracellular matrix. Fittingly, decorin (in addition to the proteoglycan biglycan, *Bgn*, and multiple collagen species) was also among the leading‐edge genes predicted to drive upregulation of the “ECM proteoglycans” gene set across multiple cell types (Figure S8).

Given the prominence of decorin, we sought to interrogate its association with complications in human diabetes. We performed a two‐sample Mendelian randomization analysis using *cis* eQTLs for decorin as instrumental variables and T1D/T1D with complications traits from the FinnGen GWAS study^33^ as outcomes (Figure 5B) and associated an eQTL‐regulating decorin transcription in fibroblasts with risk of diabetic nephropathy (Figure 5B).

### 3.6 Cellular communication indicates fibroblast activation

**FIGURE 6:**
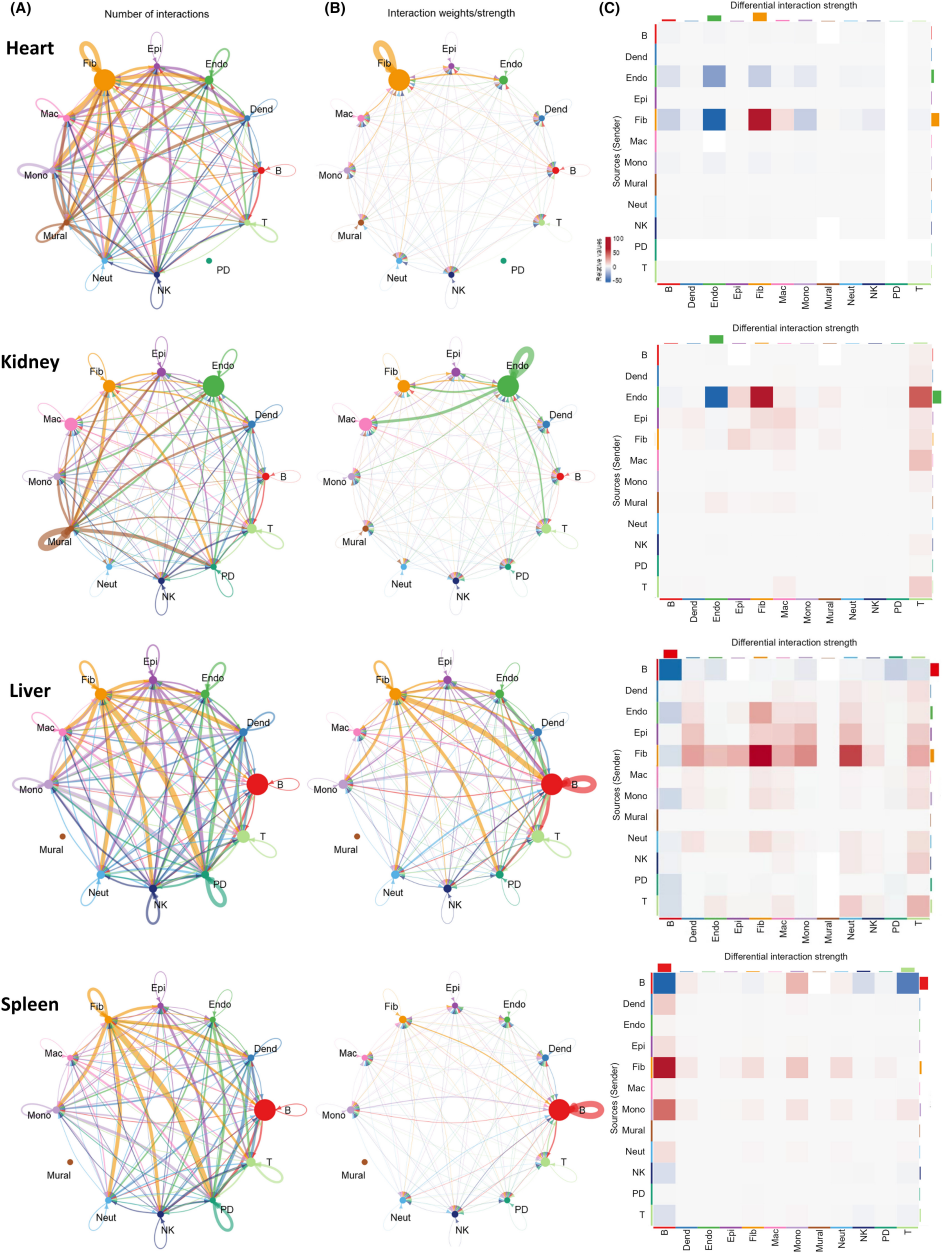
Cellular communication indicates fibroblast activation. Signaling interaction scores were evaluated by expression of ligand/receptor pairs in cells within each organ using CellChat and circle diagrams represent (A) interaction number and (B) interaction strength (adjusted according to cell number in the STZ condition). (C) The predicted strength of interactions between cell types per organ was then compared between STZ and control mice and summarized as a heatmap, with colors representing differential interaction strength. Dend, myeloid dendritic cell; Endo, endothelial cell; Epi, epithelial cell; Fib, fibroblast; Mac, macrophage; Mono, monocyte; Neut, neutrophil; PD, plasmacytoid dendritic cell.

STZ treatment resulted in perturbed gene expression across cell types and organs. To understand how these changes affect the organ‐specific interplay of immune and “non‐immune” cells, we assessed cellular crosstalk based on the expression of known ligands and receptors. We determined the number of interactions between selected cell types (Figure 6A) and the interaction strength (adjusted according to cell number; Figure 6B) using the STZ treatment data. To assess changes in cellular cross‐talk, we next compared the interaction strength between cell types of STZ‐treated and control mice (Figure 6C). We detected altered cell communication from and to fibroblasts in each organ of STZ mice. Furthermore, STZ treatment resulted in increased signaling from ECs to fibroblasts, macrophages, T cells, and ECs, likely driven by the ligand–receptor pair of Col4a2 and the plasma membrane proteoglycan, syndecan‐4 (Sdc4; Figure S9). Our interaction analysis also revealed several high‐strength signaling relationships in the liver of STZ mice, particularly from fibroblasts to most other cell types. Overall, these data support the increased activation (via cellular communication) of fibroblasts upon induction of hyperglycemia.

## 4 DISCUSSION

Diabetes and hyperglycemia lead to complications affecting multiple organs. The pathological processes underlying these complications are plausibly mediated by common cell types such as ECs, monocytes, macrophages, and fibroblasts. We hypothesized that early responses in these cells, before organ‐specific pathologies manifest, might be shared, and we reasoned that this could be important since it would suggest targets for preventive and therapeutic interventions. Combining scRNA‐seq profiling with a mouse model of type 1 diabetes and an early time point after hyperglycemia induction, we identified dysregulation of pathways of plausible relevance to the complications of hyperglycemia. However, according to the single‐cell transcriptome profiles, most cells were aligned according to organ of origin, rather than glycemic status. Fibroblasts were the exception in this regard, and we identified the following disease‐relevant features: (1) across organs, fibroblasts from hyperglycemic mice shared numerous characteristics, some of which were suggestive of a metabolically activated state. (2) We identified a high‐abundance subset of *Lyz2* ^+^ myeloid‐like fibroblasts, comprising almost half of the fibroblasts in the liver and kidney of STZ mice. (3) We found evidence of endo‐ and epithelial‐to‐mesenchymal transition and EC dysfunction. (4) Mediators of ECM remodeling, including decorin, were upregulated in many nonfibroblast cells, and Mendelian randomization based on SNPs affecting decorin levels in fibroblasts showed a role in renal fibrosis. Finally, (5) a central role for fibroblasts as early effectors in hyperglycemia was supported by modeling of ligand–receptor pairings.

A switch to activated and/or myofibroblast phenotype occurs in T2D^35^ and myofibroblasts have been linked to progression of diabetic nephropathy.^35^, ^36^ We found upregulation of some genes associated with fibroblast activation, and p53‐regulated metabolic pathways, in STZ mouse fibroblasts, particularly in the heart and liver, in addition to an increased number of high‐mitochondria fibroblasts in the STZ mouse heart. We also found that many STZ mouse fibroblasts highly expressed lysozyme 2 (*Lyz2)*, a gene previously associated with myofibroblasts originating from a myeloid lineage.^34^ These *Lyz2* ^+^ fibroblasts exhibited increased expression of the contractile marker beta‐actin, as well as monocyte (*Cd14*) and macrophage (*Cd68*) markers. Furthermore, *Lyz2* ^*+*^ fibroblasts had increased expression of genes associated with M1‐polarized macrophages, including MHC class II genes and *Il1b*, which suggests that M1 macrophages could be a source of *Lyz2* ^*+*^ fibroblasts. To support this hypothesis, we examined transcriptomes of bone‐marrow‐derived macrophages from STZ mice^8^ and found they had an upregulation of classical fibroblast markers (e.g., vimentin and *Col4a2*), and these genes were also expressed by a majority of *Lyz2* ^+^ fibroblasts in the current study. Furthermore, M1‐polarized bone‐marrow‐derived macrophages from STZ mice^8^ had upregulated *Acta2*, *Tgfb1*, and *Stat6*–genes that have been associated with transition of myeloid cells to fibroblasts.^9^ Fibroblast‐like cells and fibrocytes originating from hematopoietic (rather than mesenchymal) progenitors have been detected in multiple contexts.^37^ For example, circulating monocytes differentiate into fibrocytes and migrate to wound sites to assist in repair^38^ and macrophage‐derived fibroblast‐like cells have also been implicated as a driver of wound repair.^39^, ^40^, ^41^ In the context of diabetes, reduced conversion of macrophages to fibroblasts can impair wound repair.^39^ Conversely, dysregulation of wound repair processes is implicated in progressive fibrotic diabetic complications including diabetic nephropathy^42^ and macrophage‐derived myofibroblasts drive collagen deposition in renal^43^ and cardiac fibrosis.^44^

Interestingly, we found increased expression of collagen‐ and ECM‐related genes in other nonfibroblast cell types, including heart and liver ECs, which also had upregulated vimentin, a key marker of EndoMT/EMT. Transition of ECs to myofibroblasts has been shown to promote renal fibrosis in STZ mice.^45^ Furthermore, lineage‐tracing experiments demonstrated that at 1 month post‐STZ treatment, EC‐derived myofibroblasts are more numerous in kidney interstitium (around 10% of all myofibroblasts at 1 month, and 24% at 6 months after STZ) and this is associated with increased collagen type IV protein.^45^

The increased number of *Lyz2* ^+^ myeloid‐like fibroblasts we observed and resulting “dilutional effect” likely led to the overall reduction in mRNA of collagen‐coding genes and ECM mediators, on average, in STZ mouse fibroblasts. Collagen genes remained highly expressed in the majority of STZ mouse fibroblasts and there is likely to be a disconnect between collagen mRNA and protein levels, as post‐transcriptional regulation is important for type I collagen accumulation in the context of fibrosis.^46^ There will be an inevitable disconnect between collagen mRNA and protein levels, as STZ mice develop histopathological signs of nephropathy, including fibrosis, at the later, for example, 6‐month time points.^47^, ^48^

In addition to fibroblast transcriptional alterations, we also found several genes that were dysregulated across various cell types after STZ treatment. These included decorin, a small leucine‐rich proteoglycan that is an important regulator of fibrosis.^49^, ^50^ Decorin expression was upregulated in cells from STZ mouse heart, kidney, liver, and spleen. Previous work suggests that decorin might be produced in response to diabetes, potentially as a moderator or dampener of fibrosis. A variant allele of decorin was associated with slower progression of diabetic nephropathy in T1D patients.^51^ Furthermore, studies using decorin knockout mice found increased nephropathy/proteinuria in the STZ model.^52^, ^53^ However, in a Mendelian randomization experiment, we associated a decorin eQTL in human fibroblasts with increased risk of diabetic nephropathy, suggesting that species‐ and cell‐type‐specific effects should be considered when therapeutically targeting decorin.

We found several transcriptional alterations in STZ mice ECs that were reminiscent of pathogenic processes involved in diabetic kidney complications. Genes altered were associated with EC dysfunction, such as hypoxia/heat shock and angiogenic factors. We noted strong downregulation of heat shock protein‐encoding genes (e.g., *Hsph1*, *Hspa1a*, and *Hspb1*) and associated heat‐shock‐related Reactome pathways (e.g., “Regulation of HSF1‐mediated heat shock”). Defective responses to hypoxia and cellular stress are implicated in hyperglycemia‐induced EC dysfunction,^54^ a cellular state that can inhibit the induction of heat shock proteins which have been described as protective against hyperglycemia‐induced cellular damage.^55^ Finally, experimental hyperglycemia can enhance myelopoiesis^56^, ^57^ and specifically within the STZ model.^58^ We found upregulation of the myelopoiesis‐associated genes *S100a6*, *S100a8*, and *S100a9* ^56^, ^57^, ^58^ in STZ mouse macrophages and monocytes (and of *S100a8* in *Lyz2* ^+^ spleen fibroblasts).

There were some limitations to this study. First, the number of mice per group affected statistical power and therefore the capacity to detect differences, for example, in cell proportion between groups. Smaller groups also meant that greater sensitivity in detecting differential gene expression was obtained from individual cells rather than from pseudo‐bulk profiles. Second, the cell types captured were not necessarily a precise reflection of the cellular makeup of tissues in vivo. Third, STZ has been suggested to have direct hepatotoxic effects including apoptosis of hepatocytes.^59^ We did not observe STZ‐induced toxic effects on hepatocytes (annotated here as epithelial cells within the liver), but detected fewer B‐cells in livers and spleens from STZ‐treated mice, which may be associated with B‐cell toxicity of STZ.^60^ Fourth, transcriptomic alterations do not always represent differences in protein level or function. Finally, as only male mice were included, further work would be needed to confirm whether these findings also translate to females.

Overall, we used an unbiased systemic approach to identify a number of cell‐type‐specific and organ‐specific genes and functional pathways as potential drivers of diabetic complications. Most notably, fibroblasts shared patterns of gene expression across organs and some expressed myeloid markers or markers of epi‐ and endo‐mesenchymal transition. Further work is needed to trace the origins of fibroblasts induced by hyperglycemia. Interestingly, the processes observed were initiated early in response to hyperglycemia and before overt organ pathology, suggesting that early intervention might be beneficial for complications of diabetes. Therefore, our findings have translational relevance, as fibrosis is a common feature of multiple organ pathologies that are associated with hyperglycemia. Based on our data, future studies will explore the mechanisms by which hyperglycemia drives cellular transition, providing potential targets for therapeutic intervention. Recent evidence from our group and others has shown that elevated blood glucose drives myelopoiesis^8^, ^56^ and induces trained immunity with sustained effects on macrophage function and pathogenicity.^8^ The current findings direct future interest toward the role of hyperglycemia in driving fibroblast activation. Finally, our study provides a rich resource for researchers to investigate the biological processes underlying early diabetic disease.

## AUTHOR CONTRIBUTIONS

Adam T. Braithwaite, Naveed Akbar, Christoph Bock, Thomas Krausgruber, and Robin P. Choudhury conceived and designed the research; Adam T. Braithwaite, Naveed Akbar, Daniela Pezzolla, Daan Paget, Thomas Krausgruber, and Ricardo Carnicer performed the research and acquired the data; Adam T. Braithwaite and Robin P. Choudhury analyzed and interpreted the data. All authors were involved in drafting and revising the manuscript.

## DISCLOSURES

The authors declare no conflicts of interest.

## Supporting information


Data S1.

Figure S1.

Figure S7.

## Data Availability

The data that support the findings of this study are openly available in the Gene Expression Omnibus at https://www.ncbi.nlm.nih.gov/geo/, reference number GSE244475.
