# Supplementary material for: Multi‐organ single‐cell RNA sequencing in mice reveals early hyperglycemia responses that converge on fibroblast dysregulation
Source: FASEB J. 2024 Feb 2;38(3):e23448. doi: 10.1096/fj.202302003R (PMC12014014; doi:10.1096/fj.202302003R)
Supplement: Supplementary file 1 — Data S1. [file FSB2-38-e23448-s002.docx]

# Supplementary figure legends

**Figure S1. Experimental mice.** Terminal measurements from n=10 streptozotocin-treated (STZ) and n=9 control mice, of which n=4 and n=3, respectively were used for single cell sequencing (sc-seq; highlighted with red points). (A) Blood glucose concentration. (B) Body mass. (C) Organ mass ratio to body mass. (D) Organ mass. * p<0.05, ** p<0.01, **** p<0.0001, Student’s t-test comparing STZ vs. control, sc-seq mice only.

**Figure S2. QC and demultiplexing.** (A–C) Scatter plots of all cells with lines illustrating QC filters for percent mitochondrial reads (percent.mt), total number of reads (nCount_RNA) and number of unique genes detected (nFeature_RNA). Doublets and negatives were determined by presence of >1, or 0, hashtag oligo (HTO) tags, respectively. (D–G) Ridge plots represent log2 ‘expression level’ (i.e. number of HTO reads) per cell, summarised per demultiplexed organ. (H) t-distributed stochastic neighbour embedding (tSNE) plot of cells clustered by HTO expression, indicating singlet clusters and intermediate doublets (with two different HTO tags, e.g. HTO-Liver_HTO-Spleen).

**Figures S3-S6. Differentially expressed genes and pathways.** Volcano plots showing differences in gene expression in key cell types (fibroblasts, endothelial cells, monocytes and macrophages), for those with >150 total cells, in heart (Figure S3), liver (Figure S4), kidney (Figure S5) and spleen (Figure S6). Points were highlighted based on adjusted p-value (p < 0.05) and fold change (log2FC > 0.5). Bar plots indicate gene set enrichment analysis of Reactome pathways within ranked genes from each cell type. Significantly enriched pathways are shown with bars indicating normalised enrichment score.

**Figure S7. Relative cell abundance.** Plots indicate relative frequency of cell sub-clusters per organ, split by disease group. Frequency of STZ vs. control mice cells was assessed by t-test. *, p<0.05; ***, p<0.001. Dend, myeloid dendritic cell; Endo, endothelial cell; Epi, epithelial cell; Fib, fibroblast; Mac, macrophage; Mono, monocyte; Neut, neutrophil, PD; plasmacytoid dendritic cell.

**Figure S8. Key enriched pathways across cell types and organs.** Key enriched Reactome pathways from gene set enrichment analysis across organs/cell types are highlighted, representing common functions for extracellular matrix (ECM), mRNA processing and heat shock. Significantly enriched pathways (adjusted p<0.05) are coloured, with colour representing normalised enrichment score (trimmed to ±2 for visualisation). Endo, endothelial cell; Fib, fibroblast; Mac, macrophage; Mono, monocyte.

**Figure S9. Upregulated ligand-receptor pairs in hyperglycaemia.** Ligand-receptor pairs were assessed by CellChat within differentially expressed genes in STZ vs. control mouse cells. Highlighted key ligand-receptor pairs are predicted to drive increased signalling from STZ mouse kidney endothelial cells. Sector width within the chord diagram represents total predicted signalling strength for an interaction. Dend, myeloid dendritic cell; Endo, endothelial cell; Epi, epithelial cell; Fib, fibroblast; Mac, macrophage; Mono, monocyte; Neut, neutrophil, PD; plasmacytoid dendritic cell.

# Supplementary methods

## 1 Identification of cell clusters and sub-clusters

Six distinct B cell clusters were identified based on expression of the markers *Cd79a* and *Cd79b* (B CD21, B Mt.Hi, B naïve 1, B naïve 2, B plasma and B spleen). One cluster expressed *Cr2* (CD21; B CD21) and two clusters had particularly high expression of *Ccr7* and *Cd83*, a marker of naïve B cells (Lüthje et al., 2008) (B naïve 1 and B naïve 2). A single cluster of plasma B cells was identified based on expression of the marker *Jchain* (Castro and Flajnik, 2014) and lower expression of *Cd79a* and *Cd79b* than in other B cell clusters (B plasma). A single B cell cluster was annotated based on high proportion (mean >10%) of mitochondrial UMIs (B Mt.Hi). Another B cell cluster was specifically identified within spleen and was designated as such (B spleen). Two clusters of dendritic cells were identified as expressing the dendritic cell marker *Itgax*. One of these clusters highly expressed *S100a4* (labelled Dend s100a4) whereas the other cluster had low expression of *S100a4* (Dend). Three highly distinct clusters were determined to be ECs from heart (Endo H), liver (Endo L) and kidney (Endo K). All expressed the EC marker *Pecam1* (Figure 1C). Cells from clusters Endo H and Endo K had high expression of *Ly6a*, while Endo L cells did not. Three clusters of epithelial cells were identified using the marker *Car2* (Epi 1, Epi 2, Epi 3). One of these specifically expressed *Car3* (Epi 2), and two expressed the markers *Cdh1* and *Epcam* (Epi 1 and Epi 3). Six fibroblast clusters specifically expressed the markers *Col1a2* and *Mmp2* (Fib, Fib act, Fib aWNT, Fib Il6, Fib Mt.Hi and Fib SCA1). One of these fibroblast clusters had particularly higher expression of *Il6* (Fib Il6). Another had relatively high expression of the mesenchymal stemness/plasticity marker *Ly6a* (Challen et al., 2009) (SCA1; Fib SCA1). Two fibroblast clusters specifically expressed the fibroblast activation marker *Postn* (Muhl et al., 2020) (Fib act and Fib aWNT) and one of these also expressed *Wif1*, a marker associated with a subset of fibroblasts that suppresses WNT signalling (Muhl et al., 2020) (Fib aWNT). One of the fibroblast clusters was determined to have a higher mitochondria content based on high percentage (mean >10%) of mitochondrial UMIs and also had some, but lower expression of *Col1a2* and *Mmp2* than other fibroblast clusters (Fib Mt.Hi). Single macrophage and Kupffer cell clusters were found to express key marker *Cd68* and *C1qa* (Mac, Kupf). Macrophages were identified based on expression of the myeloid marker *Cd68* and complement component *C1qa,* with Kupffer cells distinguished based on their specificity to liver and expression of the resident macrophage marker *Vsig4* (Li et al., 2017), while non-resident macrophages did not express *Vsig4* but specifically expressed *Ms4a7*. Three monocyte clusters expressed *Cd68* in addition to *S100a4* (Mono, Mono Ly6 1, Mono Ly6 2). Two of these clusters expressed *Ly6c*, *Ccr2* and *Chil3* (Mono Ly6 1, Mono Ly6 2). One of the *Ly6c*+ monocyte clusters was differentiated from the other based on higher expression of *Cd14* and *Il1b* (Mono Ly6 2). A mural cell cluster was identified using the markers *Acta2* (α-SMA), *Des* and *Rgs5* (Smyth et al., 2018). However, due to low number of cells in this cluster, it was not possible to determine whether these were pericytes, vascular smooth muscle cells or other mural-like cells (or a combination of these cells). A single neutrophil cluster (Neut) was determined as expressing the markers *S100a8* and *S100a9*. A single natural killer cell cluster (NK) was identified using the markers *Nkg7* (Ng et al., 2020) and *Klrb1c* (Abel et al., 2018). One cluster of plasmacytoid dendritic cells were identified as expressing the marker *Ccr9* (Wendland et al., 2007). Four T cell clusters specifically expressed the marker *Cd3d*. Of these, two distinct clusters expressed *Cd8a* (T CD8 1, and to a lesser extent, T CD8 2) and *Nkg7*, two expressed *Cd4* (T CD4, T naïve) and two expressed *Ccr7* (T naïve, and to a lesser extent, T CD8 1).

# References

Abel AM, Yang C, Thakar MS, Malarkannan S. 2018. Natural Killer Cells: Development, Maturation, and Clinical Utilization. *Frontiers in Immunology* **9**.

Castro CD, Flajnik MF. 2014. Putting J-chain back on the map: how might its expression define plasma cell development? *J Immunol* **193**:3248–3255. doi:10.4049/jimmunol.1400531

Challen GA, Boles N, Lin KK-Y, Goodell MA. 2009. Mouse hematopoietic stem cell identification and analysis. *Cytometry A* **75**:14–24. doi:10.1002/cyto.a.20674

Li J, Diao B, Guo S, Huang X, Yang C, Feng Z, Yan W, Ning Q, Zheng L, Chen Y, Wu Y. 2017. VSIG4 inhibits proinflammatory macrophage activation by reprogramming mitochondrial pyruvate metabolism. *Nat Commun* **8**:1322. doi:10.1038/s41467-017-01327-4

Lüthje K, Kretschmer B, Fleischer B, Breloer M. 2008. CD83 regulates splenic B cell maturation and peripheral B cell homeostasis. *International Immunology* **20**:949–960. doi:10.1093/intimm/dxn054

Muhl L, Genové G, Leptidis S, Liu J, He L, Mocci G, Sun Y, Gustafsson S, Buyandelger B, Chivukula IV, Segerstolpe Å, Raschperger E, Hansson EM, Björkegren JLM, Peng X-R, Vanlandewijck M, Lendahl U, Betsholtz C. 2020. Single-cell analysis uncovers fibroblast heterogeneity and criteria for fibroblast and mural cell identification and discrimination. *Nat Commun* **11**:3953. doi:10.1038/s41467-020-17740-1

Ng SS, De Labastida Rivera F, Yan J, Corvino D, Das I, Zhang P, Kuns R, Chauhan SB, Hou J, Li X-Y, Frame TCM, McEnroe BA, Moore E, Na J, Engel JA, Soon MSF, Singh B, Kueh AJ, Herold MJ, de Oca MM, Singh SS, Bunn PT, Aguilera AR, Casey M, Braun M, Ghazanfari N, Wani S, Wang Y, Amante FH, Edwards CL, Haque A, Dougall WC, Singh OP, Baxter AG, Teng MWL, Loukas A, Daly NL, Cloonan N, Degli-Esposti MA, Uzonna J, Heath WR, Bald T, Tey S-K, Nakamura K, Hill GR, Kumar R, Sundar S, Smyth MJ, Engwerda CR. 2020. The NK cell granule protein NKG7 regulates cytotoxic granule exocytosis and inflammation. *Nat Immunol* **21**:1205–1218. doi:10.1038/s41590-020-0758-6

Smyth LCD, Rustenhoven J, Scotter EL, Schweder P, Faull RLM, Park TIH, Dragunow M. 2018. Markers for human brain pericytes and smooth muscle cells. *J Chem Neuroanat* **92**:48–60. doi:10.1016/j.jchemneu.2018.06.001

Wendland M, Czeloth N, Mach N, Malissen B, Kremmer E, Pabst O, Förster R. 2007. CCR9 is a homing receptor for plasmacytoid dendritic cells to the small intestine. *Proceedings of the National Academy of Sciences* **104**:6347–6352. doi:10.1073/pnas.0609180104
