# Supplementary material for: Multi‐organ single‐cell RNA sequencing in mice reveals early hyperglycemia responses that converge on fibroblast dysregulation
Source: FASEB J. 2024 Feb 2;38(3):e23448. doi: 10.1096/fj.202302003R (PMC12014014; doi:10.1096/fj.202302003R)
Supplement: Supplementary file 2 — Figure S1. [file FSB2-38-e23448-s001.pdf]

Figure S1

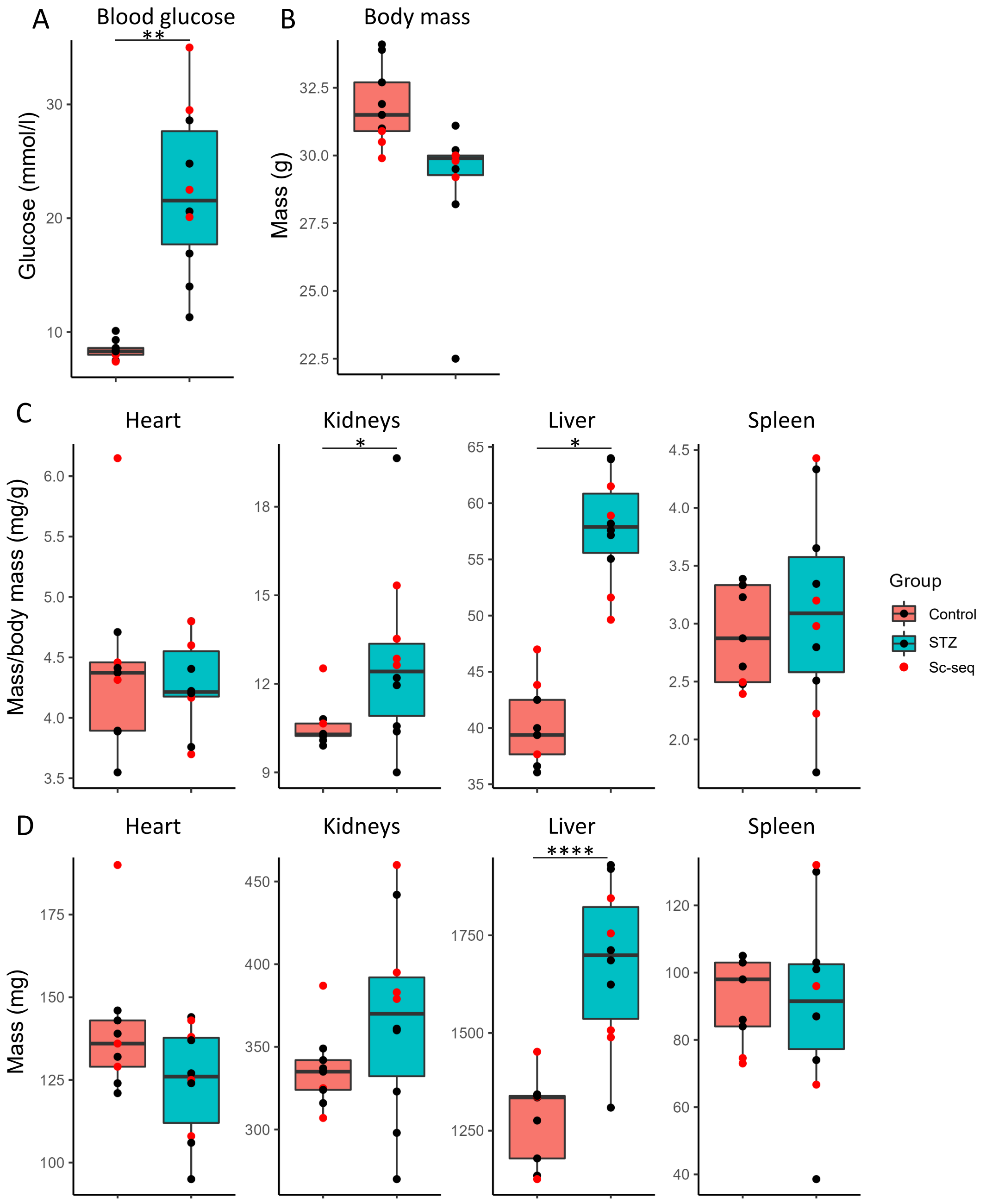
A
Blood glucose
**
Glucose (mmol/l)
30
20
10
B
Body mass
Mass (g)
32.5
30.0
27.5
25.0
22.5
C
Heart
Kidneys
*
Liver
*
Spleen
Mass/body mass (mg/g)
6.0
5.5
5.0
4.5
4.0
3.5
18
15
12
9
65
60
55
50
45
40
35
4.5
4.0
3.5
3.0
2.5
2.0
Group
Control
STZ
Sc-seq
D
Heart
Kidneys
Liver
****
Spleen
Mass (mg)
175
150
125
100
450
400
350
300
1750
1500
1250
120
100
80
60
40

Figure S2

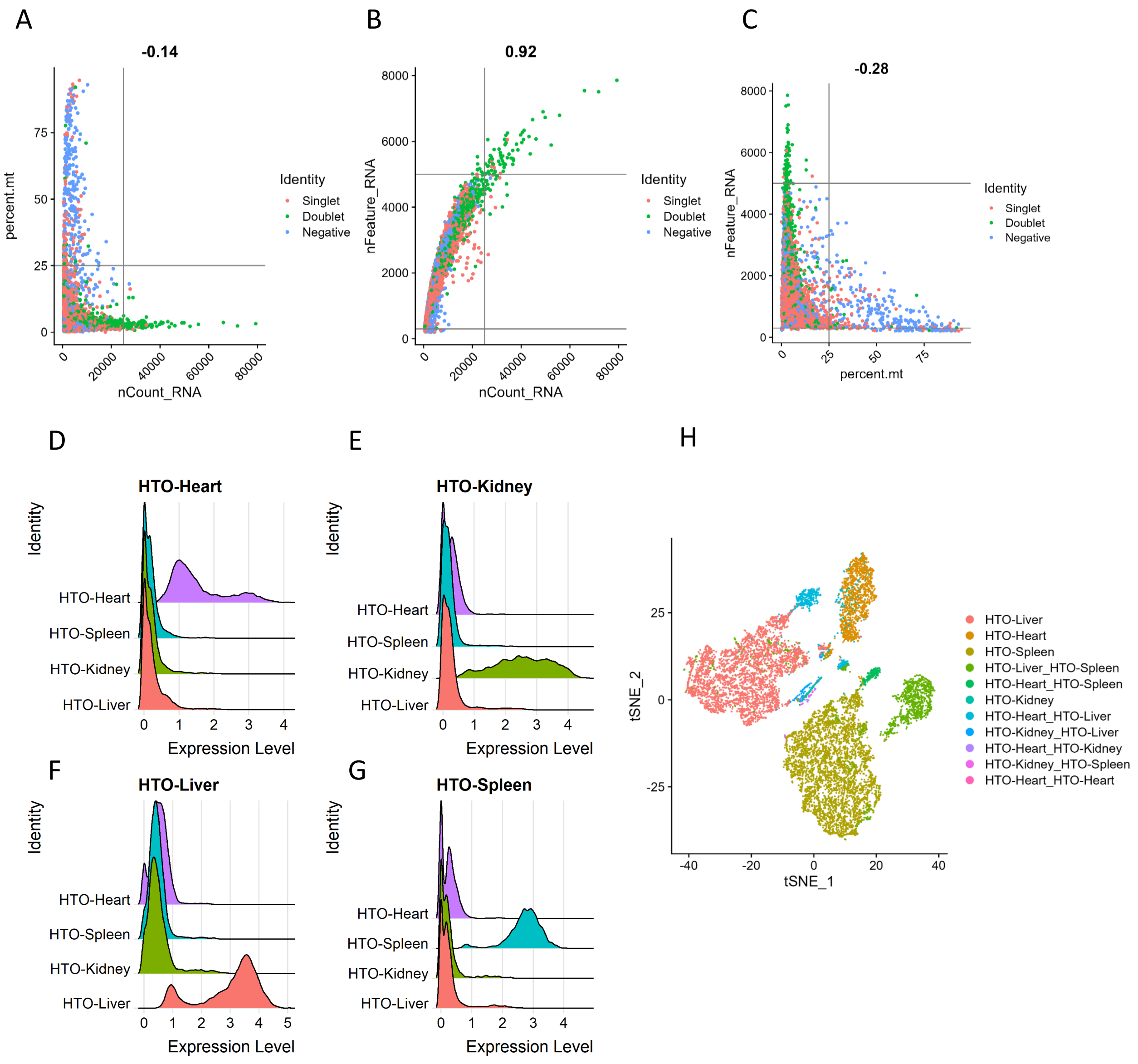

A
-0.14
percent.mt
nCount_RNA
B
0.92
nFeature_RNA
nCount_RNA
C
-0.28
nFeature_RNA
percent.mt
Identity
Singlet
Doublet
Negative
D
HTO-Heart
E
HTO-Kidney
F
HTO-Liver
G
HTO-Spleen
Identity
HTO-Heart
HTO-Spleen
HTO-Kidney
HTO-Liver
Expression Level
H
tSNE_1
tSNE_2
HTO-Liver
HTO-Heart
HTO-Spleen
HTO-Liver_HTO-Spleen
HTO-Heart_HTO-Spleen
HTO-Kidney
HTO-Heart_HTO-Liver
HTO-Kidney_HTO-Liver
HTO-Heart_HTO-Kidney
HTO-Kidney_HTO-Spleen
HTO-Heart_HTO-Heart

Figure S3

Heart

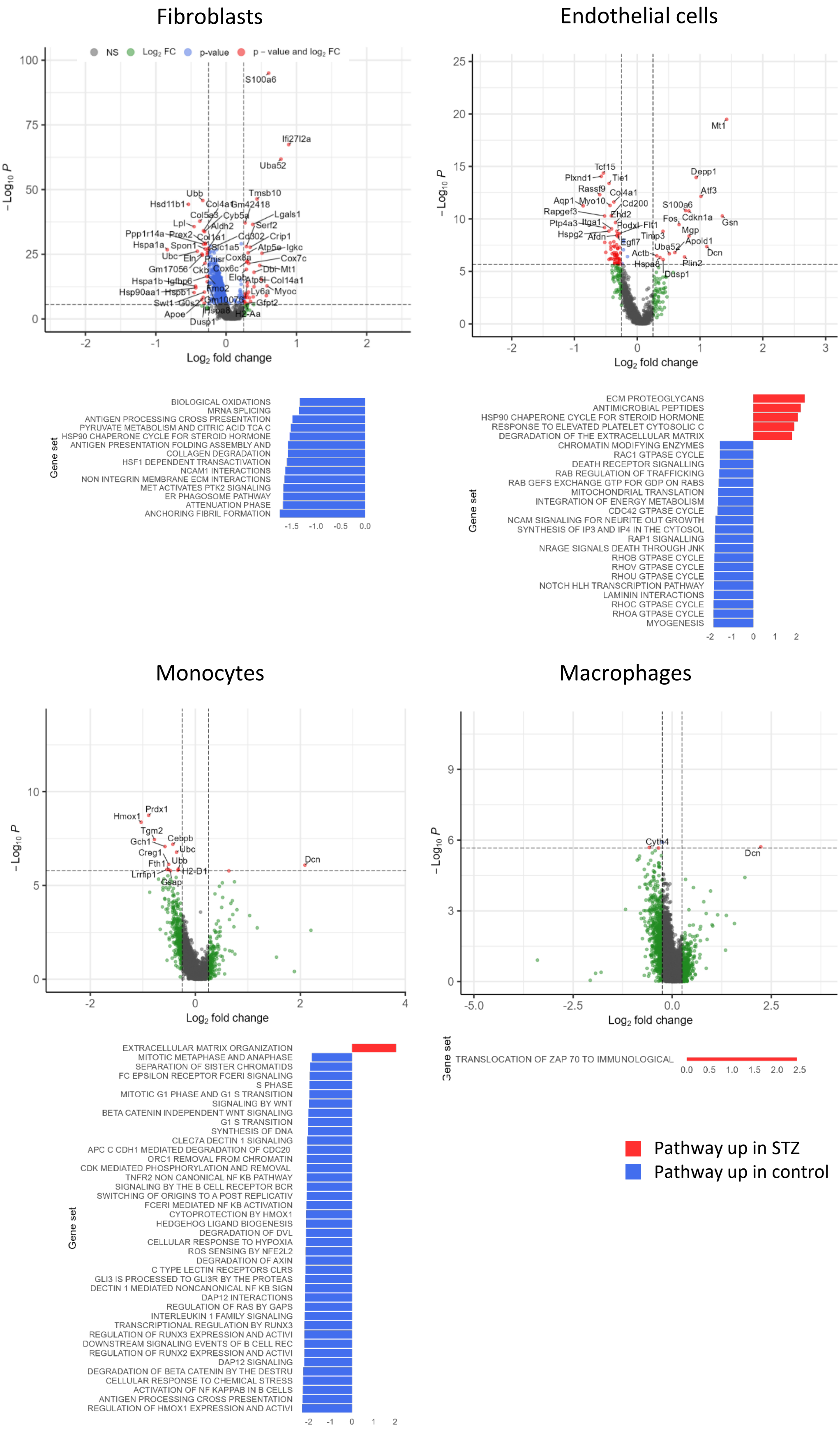

Figure S4

Kidney

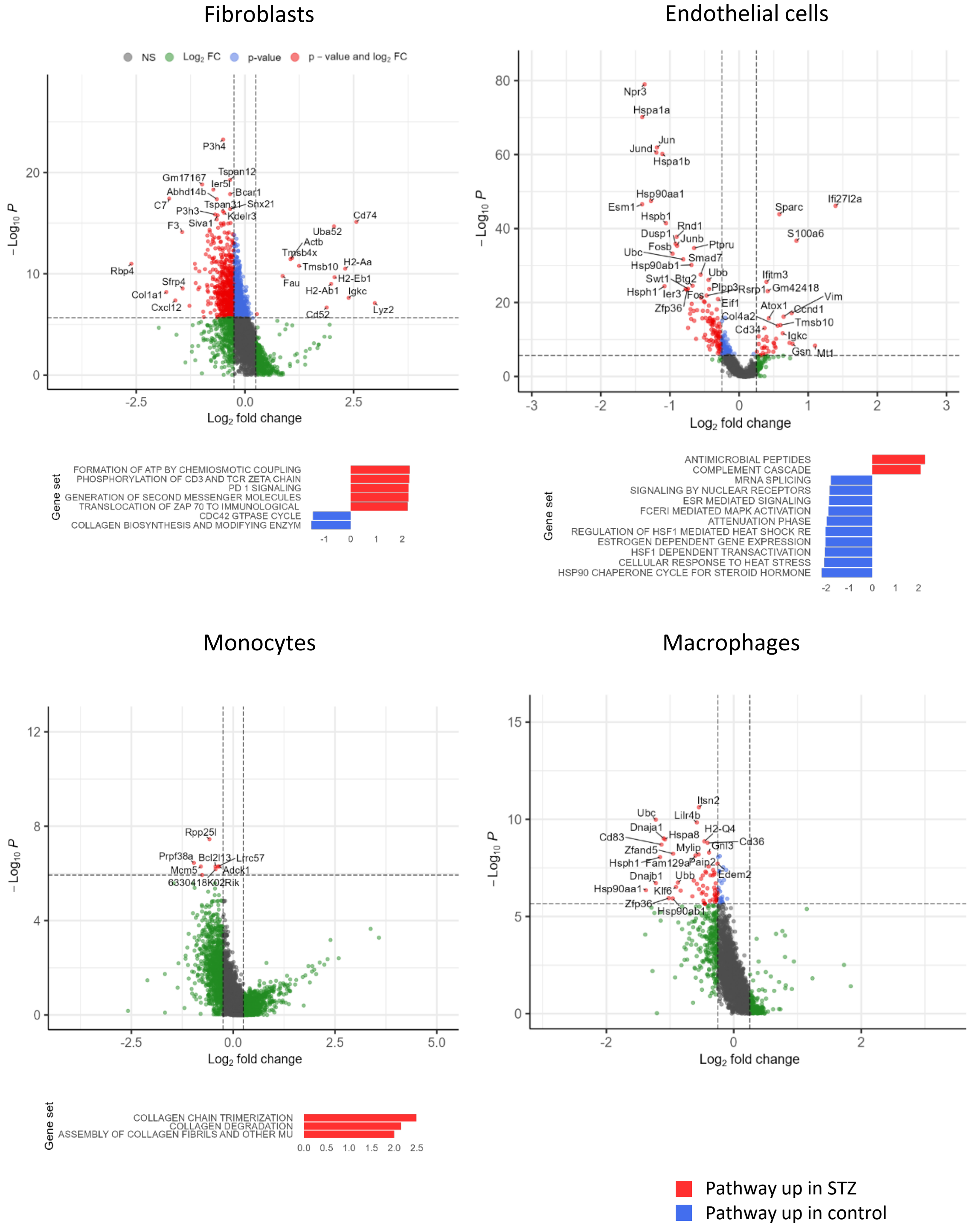

Figure S5

Liver

Fibroblasts

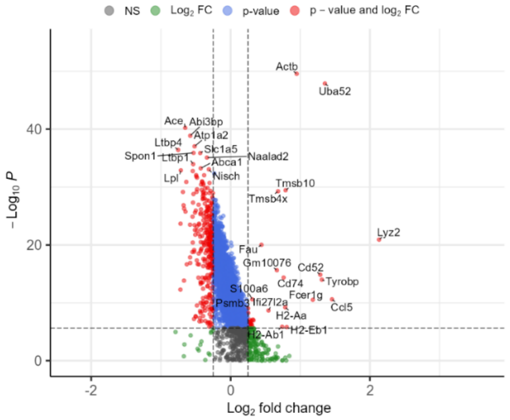

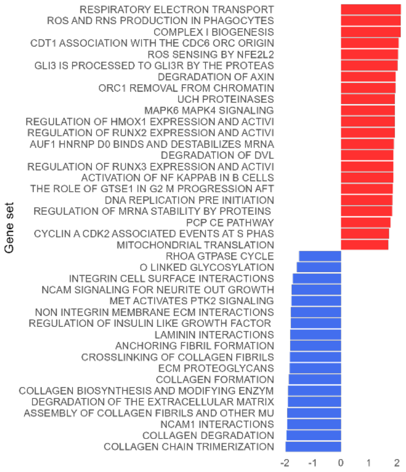

Endothelial cells

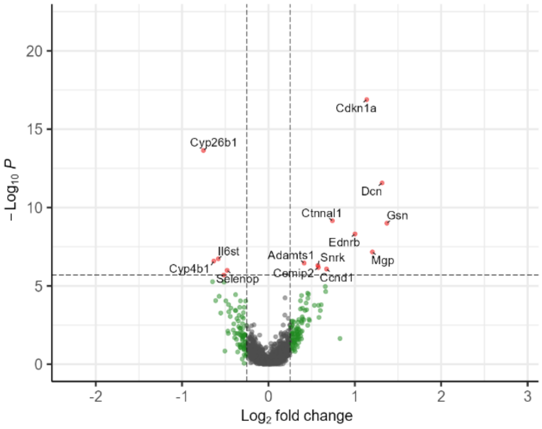

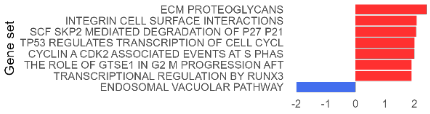

Pathway up in STZ

Pathway up in control

Monocytes

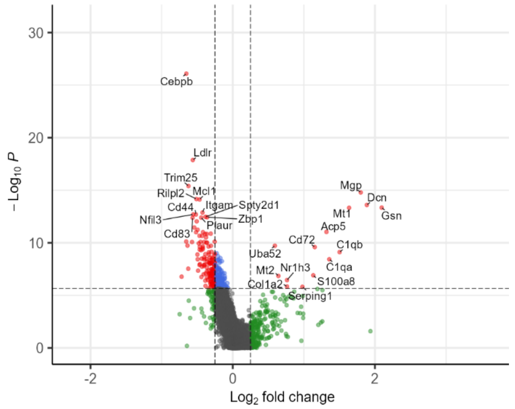

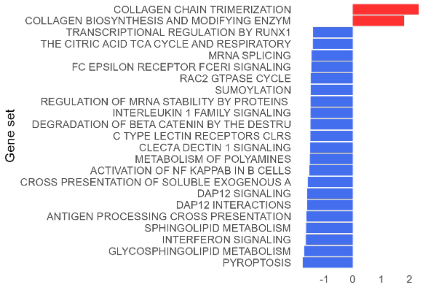

Macrophages

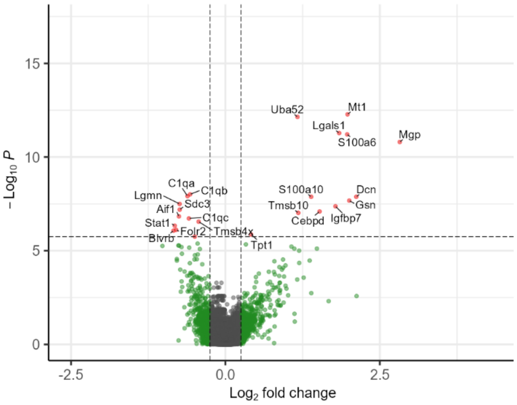

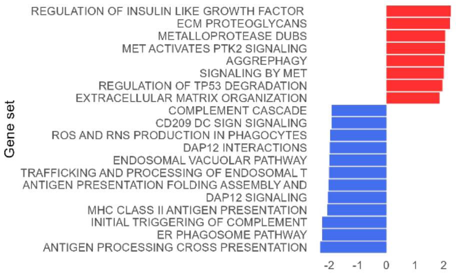

Figure S6

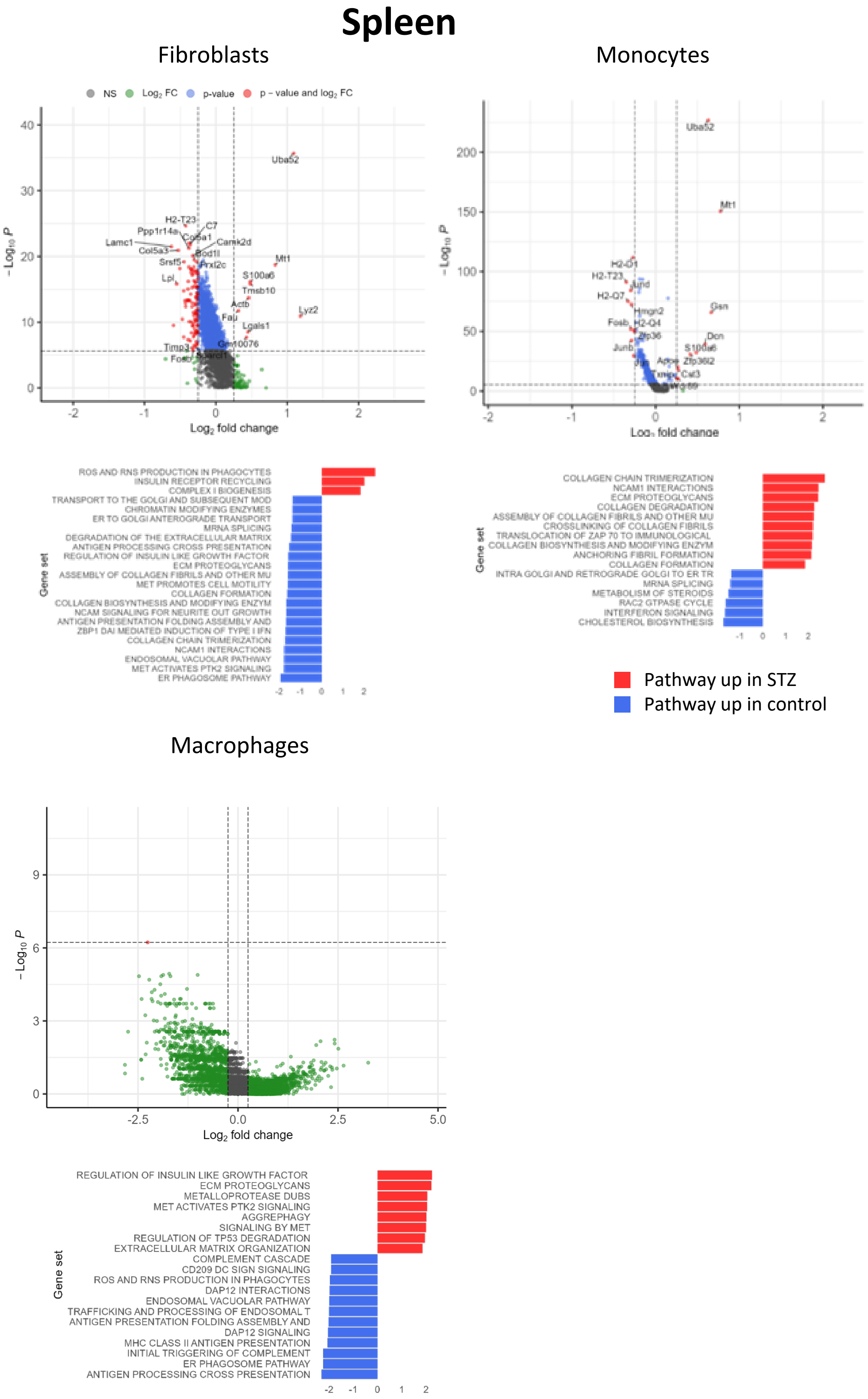

Spleen
Fibroblasts
Monocytes
Macrophages
NS
Log2 FC
p-value
p – value and log2 FC
Uba52
Mt1
Lyz2
H2-T23
C7
Ppp1r14a
Col6a1
Lamc1
Camk2d
Col5a3
Sod1l
Srsf5
Prxl2c
S100a6
Lpl
Tmsb10
Actb
Fau
Lgals1
Timp3
Gm10076
Fosb
Sparcl1
H2-D1
H2-Q7
Jund
Hmgn2
Fosb
H2-Q4
Junb
Gsn
Dcn
S100a6
Zfp36l2
Cd3
Log2 fold change
– Log10 P
Gene set
ROS AND RNS PRODUCTION IN PHAGOCYTES
INSULIN RECEPTOR RECYCLING
COMPLEX I BIOGENESIS
TRANSPORT TO THE GOLGI AND SUBSEQUENT MOD
CHROMATIN MODIFYING ENZYMES
ER TO GOLGI ANTEROGRADE TRANSPORT
MRNA SPLICING
DEGRADATION OF THE EXTRACELLULAR MATRIX
ANTIGEN PROCESSING CROSS PRESENTATION
REGULATION OF INSULIN LIKE GROWTH FACTOR
ECM PROTEOGLYCANS
ASSEMBLY OF COLLAGEN FIBRILS AND OTHER MU
MET PROMOTES CELL MOTILITY
COLLAGEN FORMATION
COLLAGEN BIOSYNTHESIS AND MODIFYING ENZYM
NCAM SIGNALING FOR NEURITE OUT GROWTH
ANTIGEN PRESENTATION FOLDING ASSEMBLY AND
ZBP1 DAI MEDIATED INDUCTION OF TYPE I IFN
COLLAGEN CHAIN TRIMERIZATION
NCAM1 INTERACTIONS
ENDOSOMAL VACUOLAR PATHWAY
MET ACTIVATES PTK2 SIGNALING
ER PHAGOSOME PATHWAY
COLLAGEN CHAIN TRIMERIZATION
NCAM1 INTERACTIONS
ECM PROTEOGLYCANS
COLLAGEN DEGRADATION
ASSEMBLY OF COLLAGEN FIBRILS AND OTHER MU
CROSSLINKING OF COLLAGEN FIBRILS
TRANSLOCATION OF ZAP 70 TO IMMUNOLOGICAL
COLLAGEN BIOSYNTHESIS AND MODIFYING ENZYM
ANCHORING FIBRIL FORMATION
COLLAGEN FORMATION
INTRA GOLGI AND RETROGRADE GOLGI TO ER TR
MRNA SPLICING
METABOLISM OF STEROIDS
RAC2 GTPASE CYCLE
INTERFERON SIGNALING
CHOLESTEROL BIOSYNTHESIS
Pathway up in STZ
Pathway up in control
REGULATION OF INSULIN LIKE GROWTH FACTOR
ECM PROTEOGLYCANS
METALLOPROTEASE DUBS
MET ACTIVATES PTK2 SIGNALING
AGGREPHAGY
SIGNALING BY MET
REGULATION OF TP53 DEGRADATION
EXTRACELLULAR MATRIX ORGANIZATION
COMPLEMENT CASCADE
CD209 DC SIGN SIGNALING
ROS AND RNS PRODUCTION IN PHAGOCYTES
DAP12 INTERACTIONS
ENDOSOMAL VACUOLAR PATHWAY
TRAFFICKING AND PROCESSING OF ENDOSOMAL T
ANTIGEN PRESENTATION FOLDING ASSEMBLY AND
DAP12 SIGNALING
MHC CLASS II ANTIGEN PRESENTATION
INITIAL TRIGGERING OF COMPLEMENT
ER PHAGOSOME PATHWAY
ANTIGEN PROCESSING CROSS PRESENTATION

Figure S8

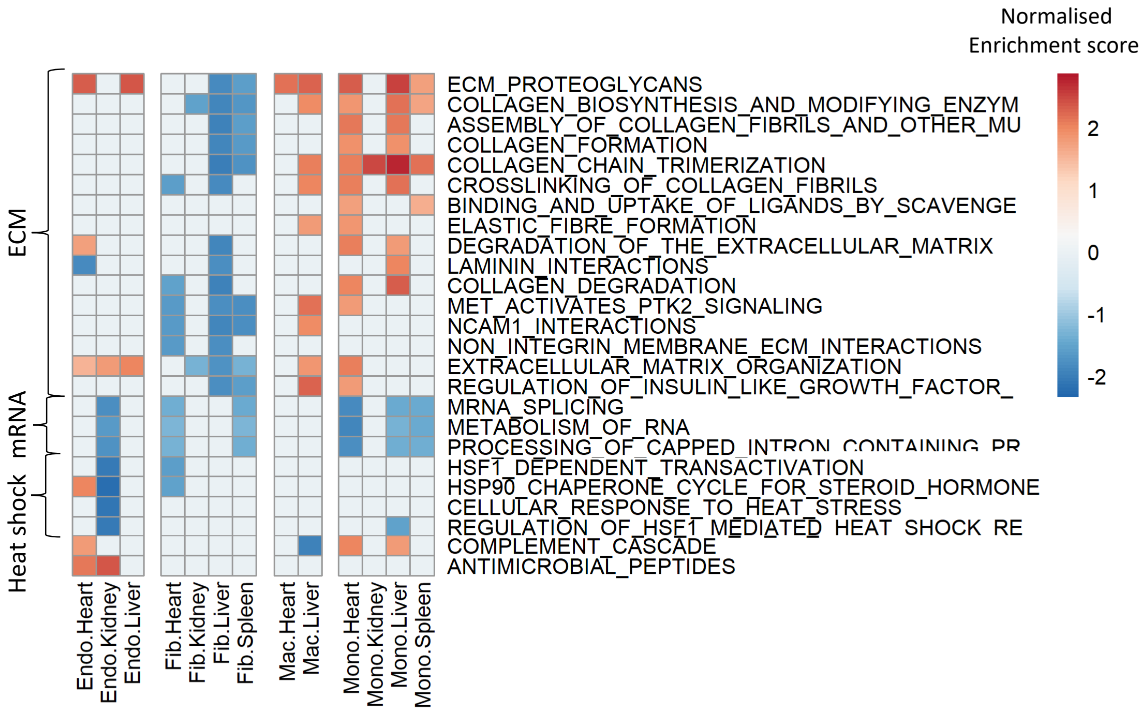
Normalised Enrichment score
2
1
0
-1
-2
ECM
mRNA
Heat shock
ECM_PROTEOGLYCANS
COLLAGEN_BIOSYNTHESIS_AND_MODIFYING_ENZYM
ASSEMBLY_OF_COLLAGEN_FIBRILS_AND_OTHER_MU
COLLAGEN_FORMATION
COLLAGEN_CHAIN_TRIMERIZATION
CROSSLINKING_OF_COLLAGEN_FIBRILS
BINDING_AND_UPTAKE_OF_LIGANDS_BY_SCAVENGE
ELASTIC_FIBRE_FORMATION
DEGRADATION_OF_THE_EXTRACELLULAR_MATRIX
LAMININ_INTERACTIONS
COLLAGEN_DEGRADATION
MET_ACTIVATES_PTK2_SIGNALING
NCAM1_INTERACTIONS
NON_INTEGRIN_MEMBRANE_ECM_INTERACTIONS
EXTRACELLULAR_MATRIX_ORGANIZATION
REGULATION_OF_INSULIN_LIKE_GROWTH_FACTOR_
MRNA_SPLICING
METABOLISM_OF_RNA
PROCESSING_OF_CAPPED_INTRON_CONTAINING_PR
HSF1_DEPENDENT_TRANSACTIVATION
HSP90_CHAPERONE_CYCLE_FOR_STEROID_HORMONE
CELLULAR_RESPONSE_TO_HEAT_STRESS
REGULATION_OF_HSF1_MEDIATED_HEAT_SHOCK_RE
COMPLEMENT_CASCADE
ANTIMICROBIAL_PEPTIDES
Endo.Heart
Endo.Kidney
Endo.Liver
Fib.Heart
Fib.Kidney
Fib.Liver
Fib.Spleen
Mac.Heart
Mac.Liver
Mono.Heart
Mono.Kidney
Mono.Liver
Mono.Spleen

Figure S9

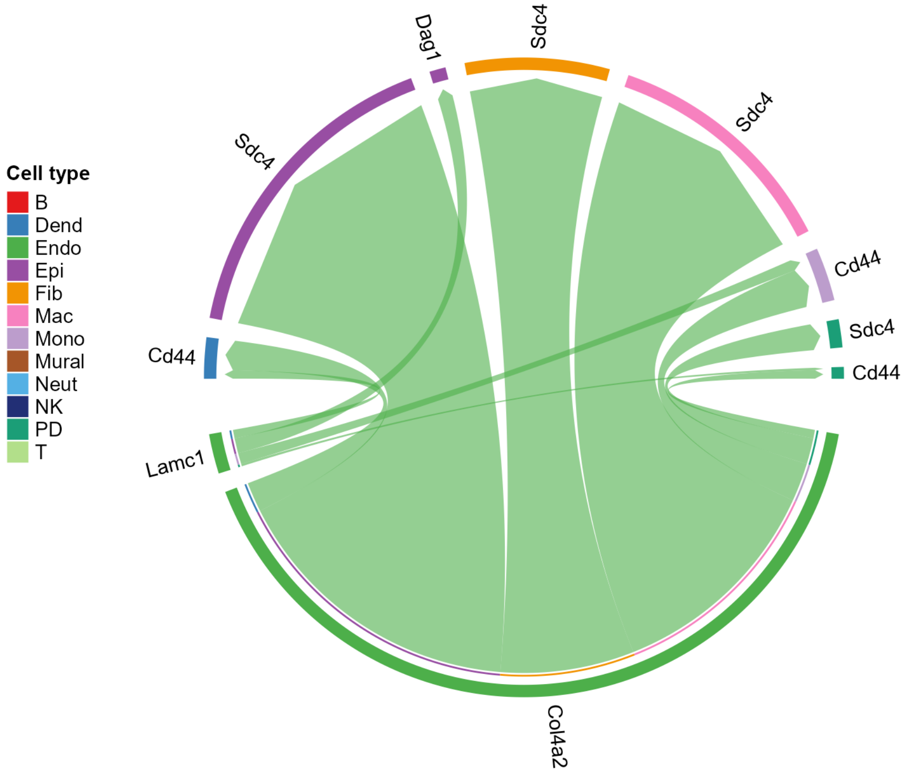

Cell type
B
Dend
Endo
Epi
Fib
Mac
Mono
Mural
Neut
NK
PD
T
Dag1
Sdc4
Sdc4
Sdc4
Cd44
Cd44
Sdc4
Cd44
Lamc1
Col4a2
