# Supplementary figures and images for: Multi‐organ single‐cell RNA sequencing in mice reveals early hyperglycemia responses that converge on fibroblast dysregulation
Source: FASEB J. 2024 Feb 2;38(3):e23448. doi: 10.1096/fj.202302003R (PMC12014014; doi:10.1096/fj.202302003R)

# Figure S7

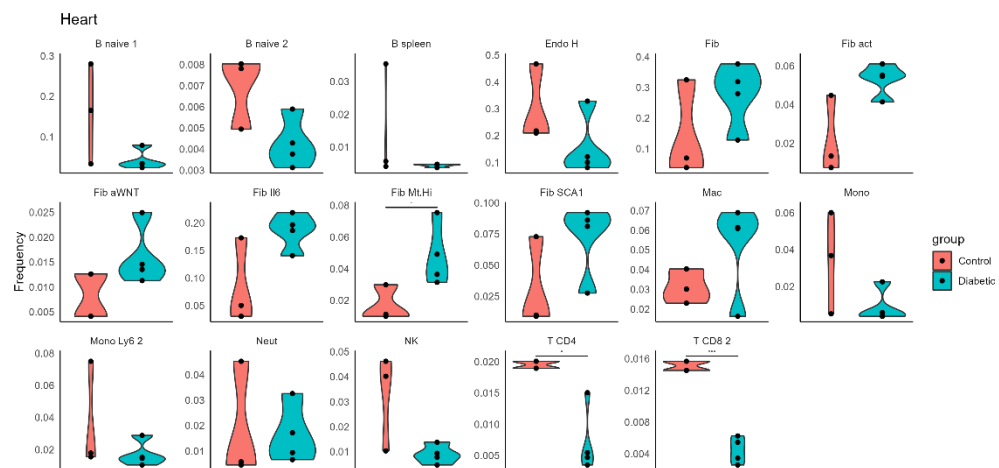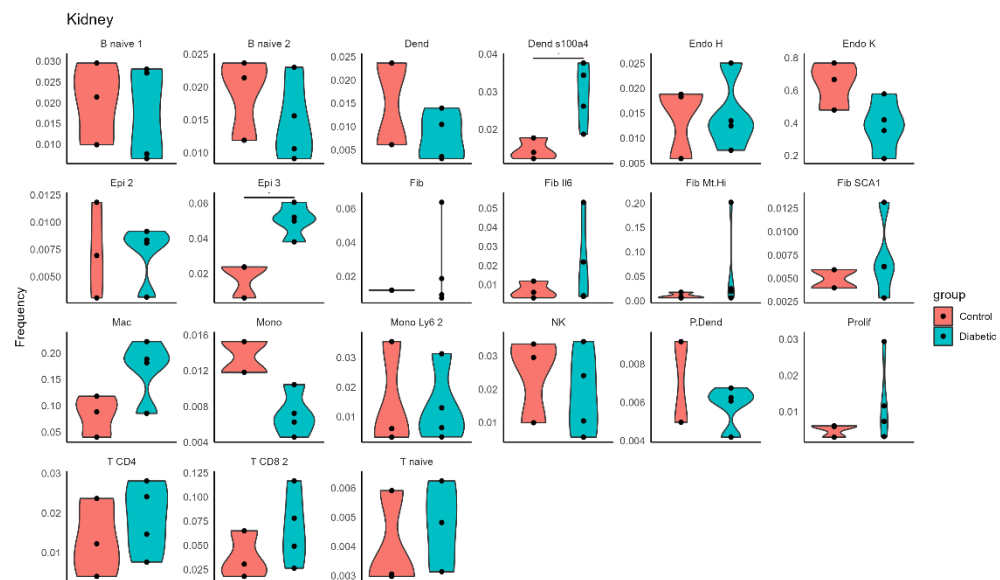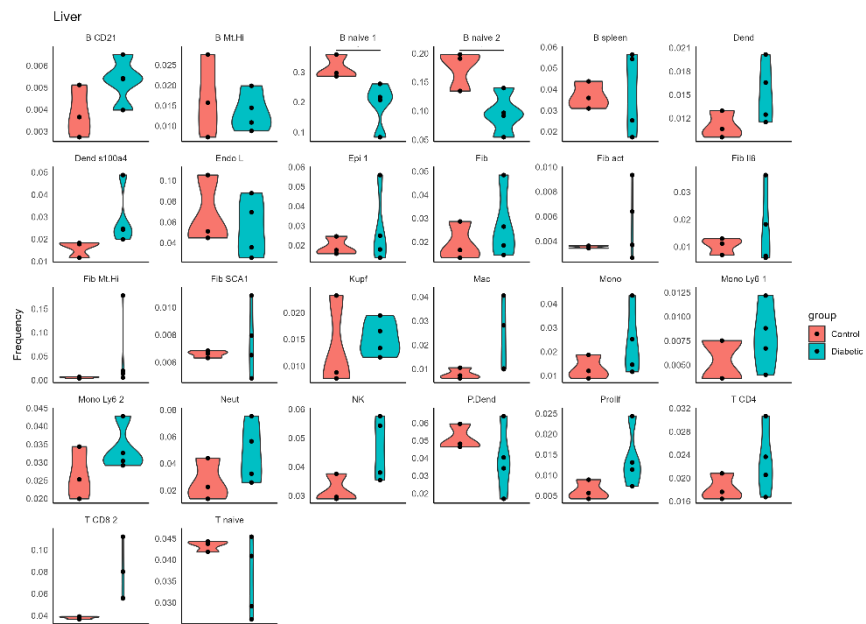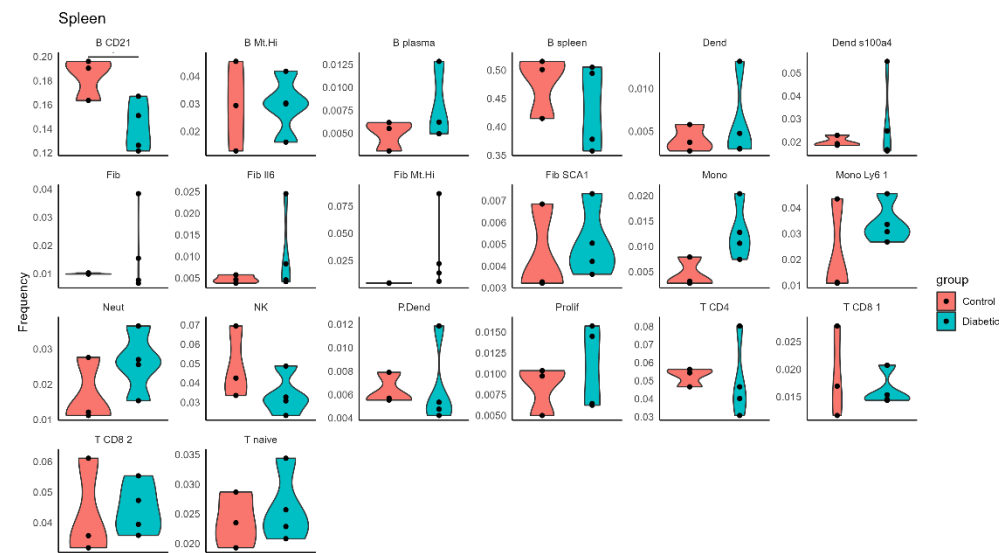

Supplement: Supplementary file 3 — Figure S7. [file FSB2-38-e23448-s003.pdf]
